# Ammonia from dinitrogen at ambient conditions by organometallic catalysts†

**DOI:** 10.1039/d2ra06156b

**Published:** 2022-11-23

**Authors:** Debashree Bora, Firdaus Rahaman Gayen, Biswajit Saha

**Affiliations:** Advanced Materials Group, Materials Sciences and Technology Division, CSIR-North East Institute of Science and Technology Jorhat Assam-785006 India bsaha@neist.res.in bischem@gmail.com; Academy of Scientific and Innovative Research (AcSIR) Ghaziabad-201002 India

## Abstract

Fixation of atmospheric dinitrogen in plants by [Mo–Fe] cofactor of nitrogenase enzyme takes place efficiently under atmospheric pressure and normal temperature. In search for an alternative methodology for the highly energy intensive Haber–Bosch process, design and synthesis of highly efficient inorganic and organometallic complexes by mimicking the structure and function of [Mo–Fe] cofactor system is highly desirable for ammonia synthesis from dinitrogen. An ideal catalyst for ammonia synthesis should effectively catalyse the reduction of dinitrogen in the presence of a proton source under mild to moderate conditions, and thereby, significantly reducing the cost of ammonia production and increasing the energy efficacy of the process. In the light of current research, it is evident that there is a plenty of scope for the development and enhanced performance of the inorganic and organometallic catalysts for ammonia synthesis under ambient temperature and pressure. The review furnishes a comprehensive outlook of numerous organometallic catalysts used in the synthesis of ammonia from dinitrogen in the past few decades.

## Abstract 2

Nitrogen fixation by organometallic catalysts at room temperature and pressure as an alternative route of Haber–Bosch process.

## Graphical abstract

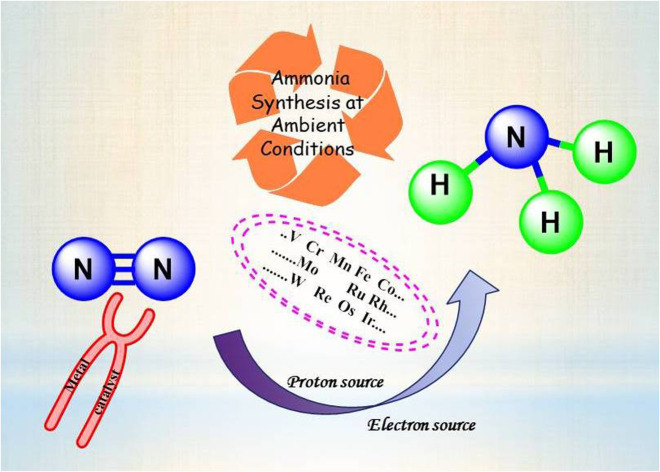

## 1. Introduction

The transformation of dinitrogen into simple nitrogenous compounds like ammonia is one of the most important processes of chemical and fertilizer industries. Ammonia solutions can directly be used as a fertilizer or can be converted to fertilizers of different chemical composition such as urea and diammonium phosphates. Ammonia is also used as building block for the synthesis of many important pharmaceutical and cleansing products and hence, ammonia production is very much essential for enduring sustainable human life on earth.^1^ Nitrogen fixation (NF) is a highly energy-demanding chemical reaction (Δ*H* = −92.28 kJ mol^−1^) due to the shorter bond length and high bond strength associated with the nitrogen–nitrogen triple bond. The inertness of molecular nitrogen can also be justified based on the stable molecular orbital electronic configuration. Fritz Haber and Carl Bosch introduced a nitrogen fixation process in 1913 that benefitted the World's growing population by providing the main industrial route to ammonia synthesis. Haber was awarded the Nobel Prize in Chemistry for ammonia synthesis in 1918. Earlier to the development of the Haber–Bosch (H–B) process, ammonia synthesis by Birkeland–Eyde and the Frank–Caro processes were proved to be highly inefficient and ammonia production was merely impossible on an industrial scale. It was through the invention of Haber and Bosch, that the ammonia production was made possible on an industrial scale which ultimately benefitted the World's growing population with artificial fertilizers ensuing adequate food production and supply. This is a fundamental redox chemical process where high pressure (200–400 atm.) and elevated temperature (400–650 °C) was used to convert dinitrogen into ammonia in the presence of hydrogen using a metal catalyst (iron/iron oxide) and a catalyst promoter (aluminium/magnesium/calcium oxides). Although, this process is the most important process to date for nitrogen fixation, it is allied with much environmental concern as it is a highly energy-intensive process and require non-renewable source like natural gas to generate hydrogen.^2^ H–B process expends more than 6% of the total energy produced in the world, and emits an enormous amount of carbon dioxide. Also, it consumes about 2% of the World's non-renewable feedstock, such as natural gas output as a hydrogen source, which can be achieved by the steam reforming process.^3–5^ Moreover, the harsh reaction condition used in this process is also not environmentally benign. From then on, with the increasing human population, nitrogen fixation has evolved in such a manner that nowadays, about 40% of the world's total population depends on H–B process for ammonia production.^6,7^ Hence, active research has been carried out to minimize the high energy consumed and also to improve the reaction conditions employed in this process. Therefore, there is a scope for optimizing the efficiency of the process by considering even moderate reduction in terms of operating temperature and pressure which will result in significant economic gain for the industrial sector.

Considering the environmental concerns related to the H–B process, intensive research has been carried out in the following fields: to improve the catalyst used to significantly lower the operating temperature and pressure, exploring the mechanism of biological nitrogen fixation system which will benefit in designing effective catalysts for ammonia synthesis under ambient conditions and, development of organometallic catalysts to explore the various methods of nitrogen fixation at ambient reaction conditions. This review evaluates the various methods of nitrogen fixation and compares their efficiency in terms of energy, environmental impacts and sustainability with the current research on this topic till date.

In order to develop an effective catalyst which could perform the energy intensive reaction at lower temperature and pressure as compared to H–B process, researchers are constantly evaluating the performance of new catalysts for ammonia synthesis. Iron catalysts in the H–B process require high temperatures of about 650–750 K and elevated pressure of 100 bars to recompense for the shift in equilibrium concentration of ammonia. As a result, very high energy is consumed in the process, along with the requirement of high equipment and gas compression costs.^3^ Ruthenium nanoparticle (Ru-NP) based catalysts were used and applied industrially instead of iron catalysts to produce ammonia in the presence of caesium (Cs), barium (Ba) and potassium (K) which acts as catalyst promoter.^8,9^ Ru-based catalysts can considerably lower the reaction temperature and pressure to 400 °C and 4–63 bar, respectively. Caesium and barium as catalyst promoters can cause substantial increase in the electron density of Ru-NPs and thus, the reaction can be carried out under less severe conditions. The promoting action of basic supports or promoters on the activity of ruthenium catalysts generally deals with the electron donating ability to the Ru-NP surfaces. When adsorption of dinitrogen occurs on the Ru crystallites, the extra electron density present in the d-orbitals of Ru atoms is donated to the antibonding orbitals of molecular nitrogen. As a result, the N–N triple bond get weakened and dissociation of dinitrogen occurs, which is considered as the rate determining step in ammonia synthesis.^10,11^ Barium (Ba) was observed to be the most active promoter although used in a smaller amount. The catalytic activity of Ru-NP catalyst can be further increased by adding a mixture of barium (Ba), caesium (Cs), and potassium (K) as promoters. The triply promoted catalyst resists methanation to a great extent and showed highest catalytic activity towards ammonia synthesis. Further studies have revealed the electronic nature of the alkali and alkaline earth metal promoters.

The efficacy of metal catalysed ammonia production can largely be increased by combining Ru with Fe catalyst, demonstrated in a small-scale reaction volume. However, Ru catalysts are susceptible to deactivation in the presence of sulphur or chlorine,^12–16^ and are also very expensive, which has restricted their use in the H–B process industrially. A pioneering work on ternary metal nitride catalyst Co_3_Mo_3_N^18^ (and control catalyst Fe_3_Mo_3_N^17^) for nitrogen fixation showed that the catalyst is two times more active (Cs used as a promoter) than the commercial iron catalyst under industrial H–B reaction conditions and sturdy enough to resist deactivation for a long period of time.^16,19–22^

**Fig. 1:**
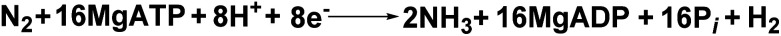
Dinitrogen reduction into ammonia by enzyme nitrogenases present in various bacteria, where Pi is inorganic phosphorous.

Another important aspect is to understand the mechanism of naturally occurring nitrogen fixation in plants. A wide variety of bacteria, such as blue-green algae, can fix nitrogen *in vivo* (natural life process) at ambient temperature and pressure. These bacteria are free living or form symbiotic associations with plants or other organisms. *Rhizobium*, *Azotobacter vinerlandii*, and *Clostridium pasteurianum* are some of the important classes of bacteria which fixes nitrogen, of which rhizobium is the best known and is found in the root nodules of leguminous plants such as clover, beans, peas, *etc.* Microorganisms like bacteria fix nitrogen with the help of the nitrogenase enzyme, which functions like a catalyst during the reduction of dinitrogen to ammonia. Three types of nitrogenases are known such as, molybdenum (Mo), iron (Fe) and vanadium (V) nitrogenases. Nitrogenases are composed of two metalloproteins: an iron protein and MFe (M = Mo, V, and Fe) protein as a cofactor. The most commonly known and best learned is the molybdenum nitrogenase, where the Mo centre of the cofactor serves as the N_2_ binding site and carries out the reduction of N_2_.^23^ The enzyme nitrogenases in various bacteria catalyses the activation of dinitrogen for the formation of ammonia according to the equation (Fig. 1).

**Fig. 2:**
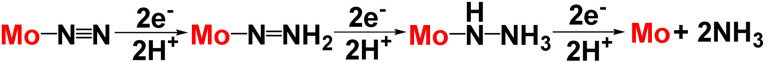
Electron transfer in Mo–Fe protein.

The electrons necessary for nitrogen reduction are transferred to nitrogen by the reduced form of ferredoxins and flavodoxins. The source of these electrons is the oxidation of pyruvate. The electrons are first transferred to a smaller protein (Fe protein or P-cluster). The reduced Fe protein transfers its reducing electron to the Mo–Fe protein and then to the nitrogen attached to the Mo atom. A series of such electron transfer steps are (Fig. 2):

The energy for this electron transfer process is provided by the hydrolysis of ATP (adenosine triphospate) to ADP (adenosine diphospate) and inorganic phosphorous (P_i_). While investigating the mechanism of the biological nitrogen fixation system, researchers were interested in marking it as a paradigm for a method that can substitute the H–B process at room temperature.

Various catalytic systems were designed to mimic the biological fixation of molecular nitrogen. Different transition metals were employed, and their dinitrogen complexes were designed and synthesized to achieve a stoichiometric amount of ammonia under mild reaction conditions. But there are only a few examples for catalytic conversion of a molecular N_2_ using these transition metal catalysts to produce ammonia under atmospheric pressure and room temperature. Shrock and co-workers in 2003 reported a molybdenum–N_2_ complex bearing a tetradentate ligand named triamidomonoamine that catalyzed the reduction of dinitrogen into ammonia. Less than 8 equiv. of NH_3_ was obtained as per the catalyst.^24^ Another successful example to catalytically convert dinitrogen was reported by Nishibayashi and co-workers, where an N_2_-bridged complex bearing two molybdenum atoms with tridentate PNP-based pincer ligands was used as a catalyst. They could successfully achieve ammonia up to 23 equiv. based on their catalyst (12 equiv. ammonia per molybdenum atom) at ambient conditions.^25^ More recently, Peters and co-workers described a tris(phosphine)borane-supported iron complex that catalyses the direct transformation of N_2_ into NH_3_ at a temperature of −78 °C.^26^ More than 40% of protons, as well as reducing equivalents, were supplied to N_2_ in their case, and 7 equiv. of ammonia could be obtained by the anionic Fe–N_2_ complex consisting of tris(phosphine)borane. This review covers almost all aspects of Schrock, Nishibayashi, Peters, and Chirik type catalysts for N_2_ fixation under mild conditions.

## 2. Early work

### 2.1. Chatt's work

**Fig. 3:**
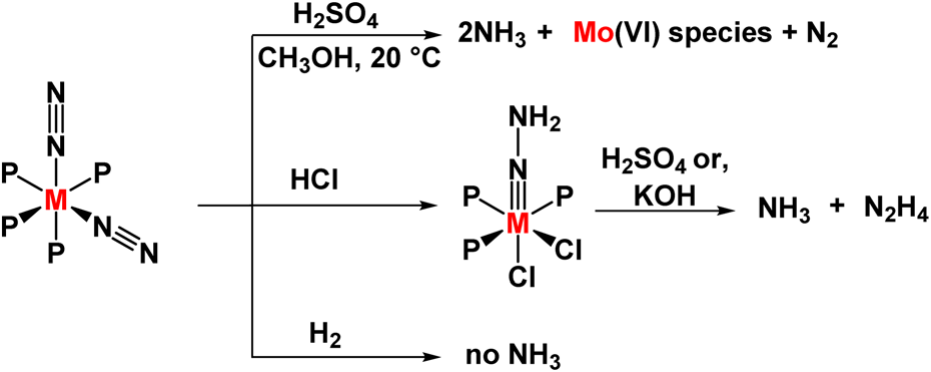
Group 6 (M = Mo, W) dinitrogen complexes towards ammonia synthesis.

Homogeneous ammonia production was explored by Chatt and Hidai in the 1960s, where they synthesized group 6 metal complexes with bidentate phosphine ligands like dppe (bis(diphenylphosphino)ethane) and depe (bis(diethylphosphino)ethane) (Fig. 3).^27^ The reaction of the *cis*-[M(N_2_)_2_(PMe_2_Ph)_4_] complex and *trans*-[M(N_2_)_2_(PMePh_2_)_4_] complex (where M = Mo, W) with sulphuric acid in methanol as solvent at 20 °C gives 1.9 and 0.7 NH_3_ per W atom and Mo atom respectively, along with a minimal amount of hydrazine for (M = W). The mechanism involved in these reactions is relevant to the action of nitrogenase, *i.e.*, biological nitrogen fixation. From there two points can be considered – (1) in the presence of monotertiary phosphines, dinitrogen reduction to ammonia can be achieved at room temperature (2) oxygen-containing solvents or oxo-anions facilitate the reduction.

A clear view of the reaction pathway can be obtained by isolating the intermediates formed and identifying the diazenido, hydrazido, and hydrazinium complexes attained from the dinitrogen complexes. Also, the other complexes like nitride (MN), amido (MNH_2_), imido (MNH), and ammine (MNH_3_) formed were isolated and identified. These intermediates ultimately led to the formation of a catalytic amount of ammonia, and this cycle was named the Chatt cycle,^28^ which runs between oxidation states, Mo(0) and Mo(iv). All the electrons required for catalytic conversion into ammonia were provided by the zero valent Mo and W metals. It is one of the pioneering examples of converting dinitrogen into ammonia at room temperature and pressure through metal-based catalysis.

Comparatively, in the biological nitrogen fixation system, hydrazine was coordinated as a reactive intermediate^29^ as is evident from the reaction mechanism but in the case of this Chatt cycle, hydrazine is obtained in a side reaction rather than in the main reaction stream.

## 3. Present development

### 3.1. Richard R. Schrock's work

**Fig. 4:**
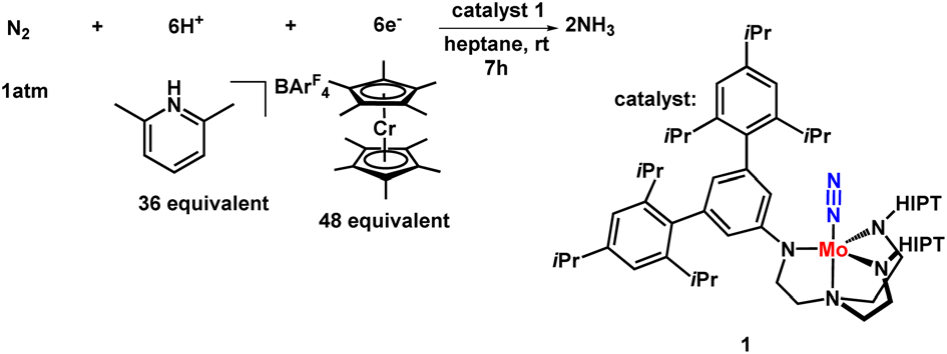
Nitrogen fixation with Mo-triamidoamine catalyst.

Shrock and Yandulov reported the first successful example in 2003 to catalytically transform dinitrogen into ammonia under ambient reaction conditions. They introduced a molybdenum-based dinitrogen catalyst having triamidoamine as a ligand consisting of a bulky substituent HIPT (hexa-iso-propyl-terphenyl) attached to it to achieve ammonia at mild reaction conditions (Fig. 4). Along with this catalyst, decamethylchromocene 
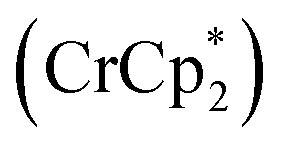
 was used as a reductant, and the proton source used here was 2,6-lutidinium tetrakis [3,5-bis(trifluoromethyl)phenylborate] ([LutH]BArF_4_) to achieve 8 equiv. of ammonia from dinitrogen based upon the catalyst at ambient temperature and pressure.^24,30^

**Fig. 5:**
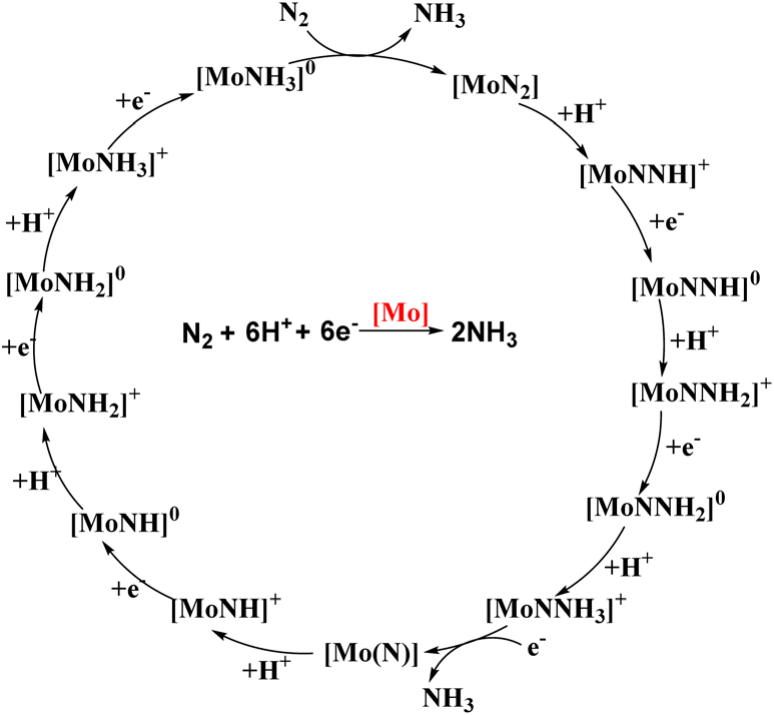
Schrock–Yandulov cycle using complex 1 as a catalyst.

The reaction was effectuated by the controlled step-by-step inclusion of protons and electrons without the need for molecular dihydrogen (Fig. 5), which was unique, although H_2_ was obtained as a by-product, leading to the generation of two molecules of ammonia.^30*b,c*,31^ The well-known Schrock cycle was accomplished by theoretical studies and isolation of some of the intermediates formed like diazenido, nitride, hydrido, and ammonia complexes which gave a mechanistic insight of the detailed reaction pathway. Studies were also carried out by synthesizing different complexes involving the molybdenum triamidoamine system,^32^ diamido pyrrolyl molybdenum complexes,^33^ [(DPPNCH_2_CH_2_)_3_N]^3−^ molybdenum complexes where DPP stands for 3,5-(2,5-diisopropylpyrrolyl)2C_6_H_3_)^34^ and investigation of these catalysts towards catalytic dinitrogen reduction were screened. Electrochemical studies were performed for N_2_ reduction using [HIPTN_3_N]Mo complexes (where HIPTN_3_N = (3,5-(2,4,6-i-Pr_3_C_6_H_2_)_2_–C_6_H_3_NCH_2_CH_2_)_3_N) and the redox properties of the intermediates in the catalytic cycle were discussed.^35^ Then again, Schrock and his co-workers in 2017 developed and synthesized molybdenum diamido complexes and achieved up to 10 equiv. ammonia per Mo atom.^36^ On the contrary, vanadium (V) and other group 6 transition metals, tungsten (W) and chromium (Cr), gave stoichiometric conversion of ammonia.^37^

### 3.2. Y. Nishibayashi's work

**Fig. 6:**
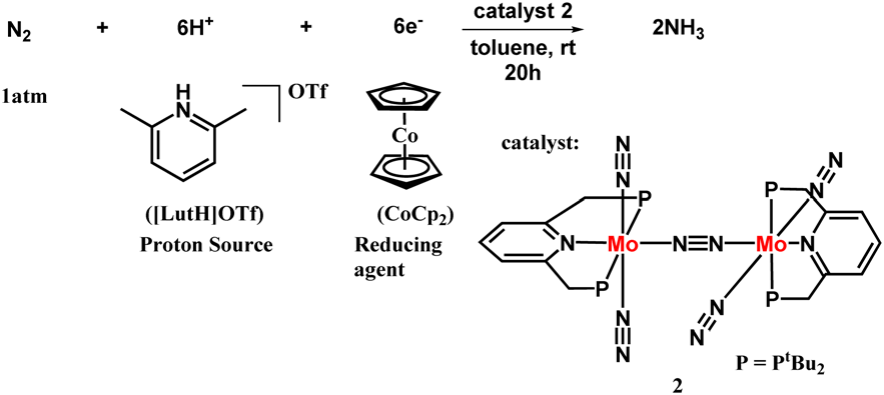
Dimolybdenum PNP pincer complex catalyzed nitrogen fixation.

The next successful example of the catalytic ammonia synthesis after the Schrock group was reported in 2010 by Nishibayashi and his co-workers. Thus, a complex having a dinitrogen bridged dimolybdenum system has been proposed consisting of a tridentate PNP-based pincer ligand for the production of ammonia at room temperature and pressure (Fig. 6). A milder reducing agent *i.e.*, cobaltocene, and a proton source namely, [LutH]OTf was used along with a catalytic amount of the Mo catalyst and 23 equiv. ammonia was obtained based upon the catalyst (12 equiv. acquired per Mo atom).^25^ To favour the formation of ammonia, the nature of the proton source and reductant should be taken into account. A milder reductant with high reducing ability along with a proton source with suitable acidity is essential for the effective change of dinitrogen to ammonia. On the other hand, different Mo-complexes were prepared to check the feasibility of this reaction which concluded the formation of ammonia in less than a stoichiometric amount. Only the dimolybdenum complex possessing tridentate PNP kind of pincer ligand, *i.e.* [{Mo(N_2_)_2_(PNP)}_2_(μ-N_2_)], was efficacious in ammonia formation from dinitrogen.

**Fig. 7:**
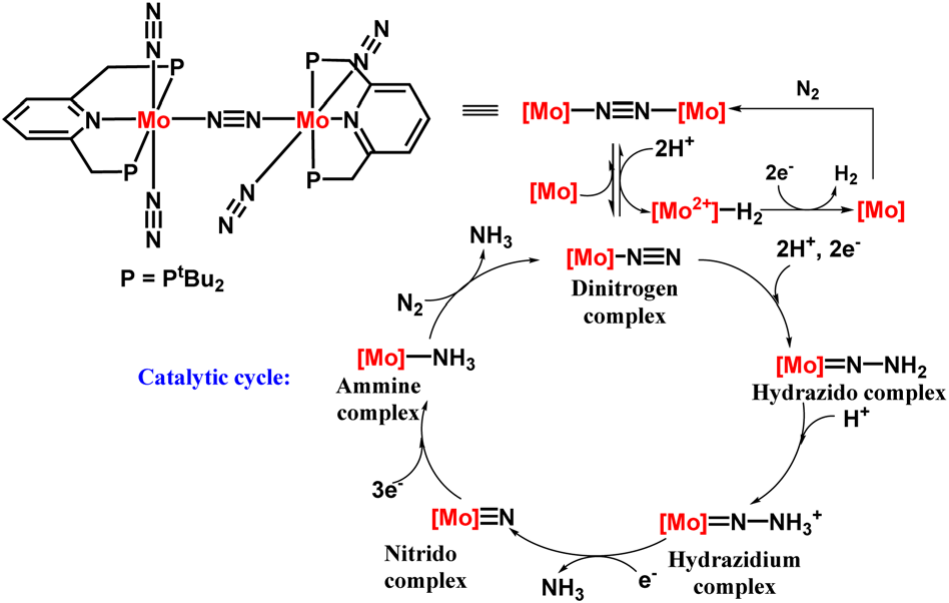
Proposed reaction pathway with PNP-type pincer dimolybdenum system.

A catalytic cycle in the synthesis of ammonia from dinitrogen was proposed, relying on the catalytic and stoichiometric reactions (Fig. 7). In the initiation step, protonation occurs on one of the molybdenum atoms in the catalyst leading to a catalytically active mononuclear dinitrogen species and an inactive hydride complex. After that the dinitrogen species was protonated to afford a hydrazidium molybdenum complex by means of a hydrazido complex, wherein the bond between the two nitrogen atoms gets cleaved releasing ammonia and a nitride complex altogether. Successively, the nitride complex upon reduction and protonation, gives the ammine complex that gives another ammonia molecule along with the dinitrogen complex regenerating the starting complex. Dihydrogen is also produced by reducing the hydride complex obtained in the initiation step.

Although the appropriate reason why this PNP-type pincer ligand containing complex is more efficient is not adequately understood, yet it can be assumed that the way of coordination of the pincer ligand in a meridional configuration to the metal center forefronts the stability of the binding site for dinitrogen transformation.^3^

**Fig. 8:**
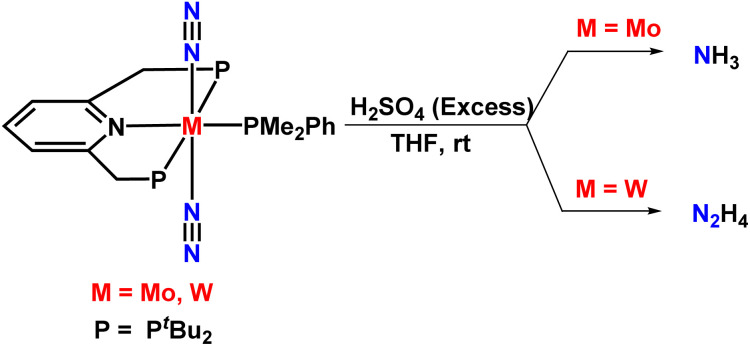
Molybdenum (Mo), tungsten (W) dinitrogen complexes in the production of ammonia and hydrazine respectively.

Nishibayashi's group further investigated the formation of ammonia by reporting different novel Mo and W dinitrogen complexes having PNP-based pincer ligands in 2012. Both these complexes, on treatment with an excess amount of sulphuric acid, yielded ammonia and hydrazine at room temperature (Fig. 8). A dinitrogen-bridged dimolybdenum carbonyl complex was also prepared from the previously reported dinitrogen-bridged dimolybdenum complex, but unfortunately this complex gave a lower yield of ammonia. In the case of the tungsten complex, 0.62 equiv. hydrazine could be achieved based on W atom along with 0.17 equiv. NH_3_ based on the W atom. It could be understood that the protonation steps of the dinitrogen complexes are affected by the nature of the metal incorporated in them and by the type of solvents, ligands and acids used in the reaction process.^38^

After this, they tried to figure out why the dinitrogen bridged PNP-based dimolybdenum complex performs as a better catalyst than the monometallic dinitrogen complexes. Hence, the outset in the catalytic performance of dinitrogen-bridged dimolybdenum catalyst supported by pincer ligand has been investigated with DFT calculations with respect to stoichiometric and catalytic production of ammonia under mild conditions. Nishibayashi *et al.* in 2014 reported a plausible reaction mechanism based on both experimental and theoretical learnings. According to their study, a synergy exists between the Mo atoms linked to a dinitrogen bounded ligand while protonating the coordinated N_2_ ligand.^39^ One of the molybdenum atoms donates one electron *via* bridging dinitrogen ligand to the other molybdenum core's active site, thus making a terminal dinitrogen ligand ready to accept a proton at their active site. One metal center containing the PNP-based pincer ligand acts as a mobile electron carrier ligand to the other metal core at their active site. These findings were indifferent to the usual part played by the dinuclear N_2_-bridged metal complexes possessing PNP-based and PCP-based pincer ligands. These complexes were observed to be employed as precursors to the reactive mononuclear species.^40–42^

Then again, research was continued regarding the dimolybdenum dinitrogen bounded complex with a PNP-supported ligand to investigate whether the introduction of any substituent in the ligand of this complex would bring about any effect or change in the catalytic activity towards ammonia formation. In 2014, the same group has reported that introducing methoxy group in the 4^th^ position of PNP aided ligand system in the complex works best as a catalyst in dinitrogen reduction to ammonia. Dihydrogen was formed as by-product that was complementary to the ammonia production in the reaction system. 52 equiv. ammonia was obtained based upon the catalyst (up to 26 equiv. ammonia achieved per molybdenum atom). Electron donating groups present in the ligand not only affect the electronic environment but also boost the protonation steps during the initial protonation step in the catalytic transformation of dinitrogen into ammonia.^43^

**Fig. 9:**
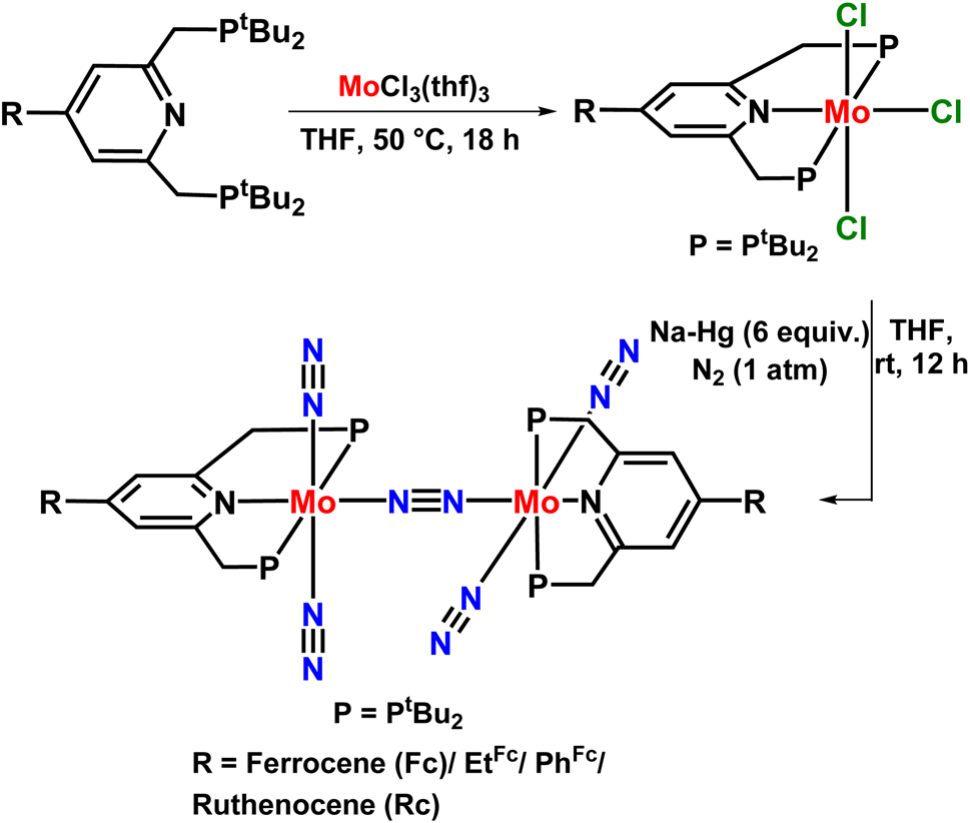
Dimolybdenum dinitrogen catalyst with a redox-active PNP-based pincer ligand towards nitrogen fixation.

In 2015, Nishibayashi explored the catalytic activity of the previously reported PNP-based pincer ligand by introducing a redox-active moiety ferrocene like ferrocenyl (Fc), 4-ferrocenylphenyl (PhFc), 2-ferrocenylethyl (EtFc), and ruthenocenyl (Rc), *etc.* to the 4^th^ position of the pyridine ring (Fig. 9). This redox-active moiety enhances the reduction process by transferring electrons intramolecularly from the iron atom of ferrocene moiety into the active site of the Mo atom of the dimolybdenum dinitrogen complex, thus accelerating its catalytic activity towards nitrogen fixation. Thus, the complex [{Mo(N_2_)_2_(4-Fc-^*t*^BuPNP)}_2_(^1^μ-N_2_)] was proved to be the most active and efficient catalyst for the production of ammonia where 37 equiv. ammonia was attained as per the catalyst (19 equiv. based upon each Mo atom). When the proton source and reducing agent were used in a greater amount (228 equiv. 
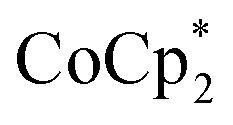
 and 384 equiv. [LutH]OTf, respectively) the amount of ammonia produced was 45 equiv., 22 equiv. based upon apiece Mo atom.^44^

Therefore, the presence of the redox-active moiety seemed to play a pivotal role by providing electrons to the metal and increasing the oxidation state on the Mo atom. Henceforth, a conclusion could be made that the presence of electron releasing groups in the pyridine ring of the complex accelerates protonation of the dinitrogen and introducing any redox active group into the pyridine ligand accelerates the reduction steps in the catalytic alteration of dinitrogen to ammonia.

**Fig. 10:**
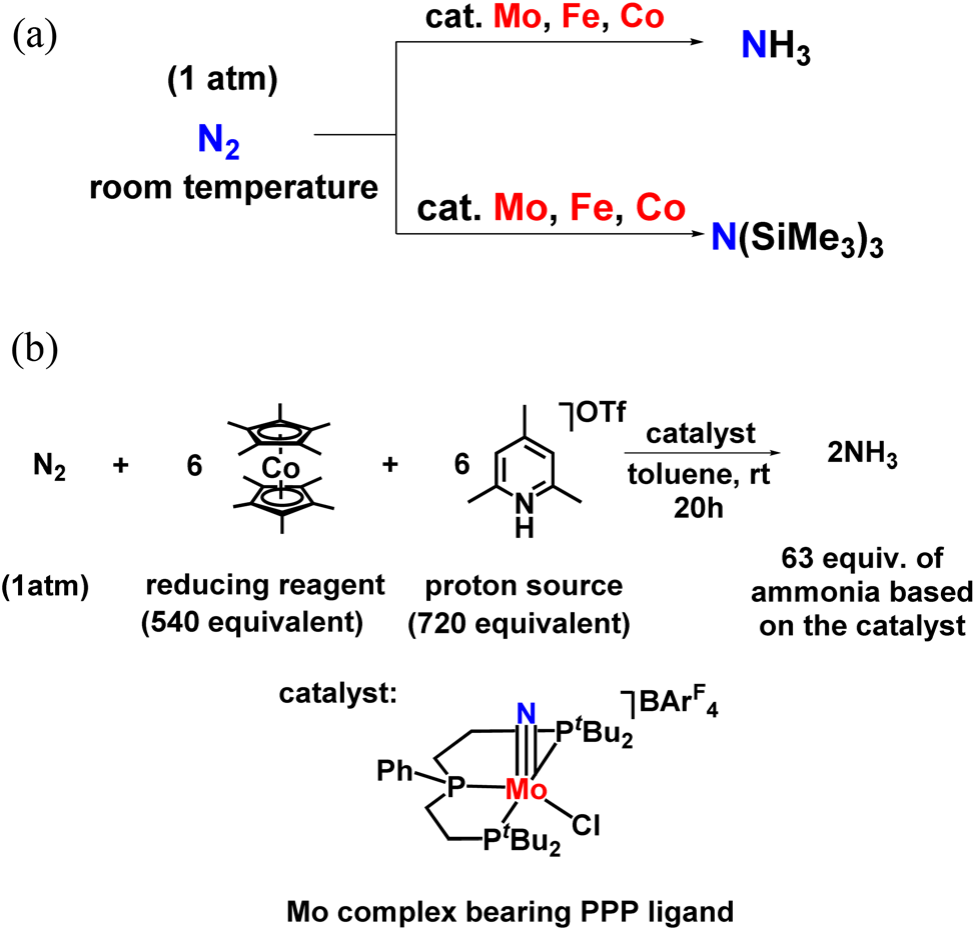
(a) Ammonia or silylamine formation from dinitrogen using Mo, Fe, and Co catalyst at ambient reaction condition. (b) Catalytic nitrogen fixation using a molybdenum catalyst bearing PPP ligand.

Catalytic dinitrogen reductions using other transition metals complexed with dinitrogen ligand as catalysts were realized and hence along with Mo, Fe and Co systems were used. It confirmed ammonia and silylamine formation under mild reaction conditions or at low temperatures (Fig. 10a). Especially in the case of the molybdenum–nitrido complex consisting of PPP ligand, 63 equiv. NH_3_ could be achieved for one Mo atom in the catalyst at ambient reaction conditions (Fig. 10b).^45^ For the formation of silylamine, atmospheric molecular dinitrogen (N_2_) was reacted with Me_3_SiCl using Na as the reducing agent along with the catalyst to catalytically change N_2_ into N(SiMe_3_)_3_ under ambient reaction conditions.

**Fig. 11:**
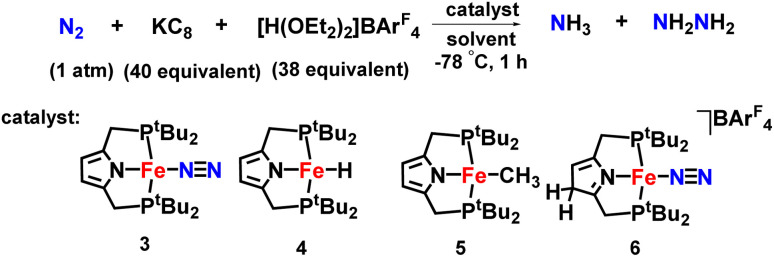
Fe complexes having PNP pincer ligand towards ammonia formation.

An iron-dinitrogen catalyst having an anionic pincer ligand, ([Fe(N_2_)(PNP)]) was reported in 2016, which worked as an efficient catalyst to convert dinitrogen to form ammonia and hydrazine catalytically (Fig. 11). An important intermediate formed in the naturally occurring biological nitrogen fixation is hydrazine. Thus, this work gives a mechanistic insight into the formation of a catalytic amount of hydrazine from dinitrogen at ambient conditions with the help of well-established iron-dinitrogen catalysts. KC_8_ was used as a reducing agent and a proton source, [H(OEt_2_)_2_]BArF_4_ was used in the reaction, and when a large amount of reducing agent and also proton source were used along with the catalyst with Et_2_O as a solvent at −78 °C, 14.3 and 1.8 equiv. of ammonia and hydrazine were obtained, respectively.^46^

**Fig. 12:**
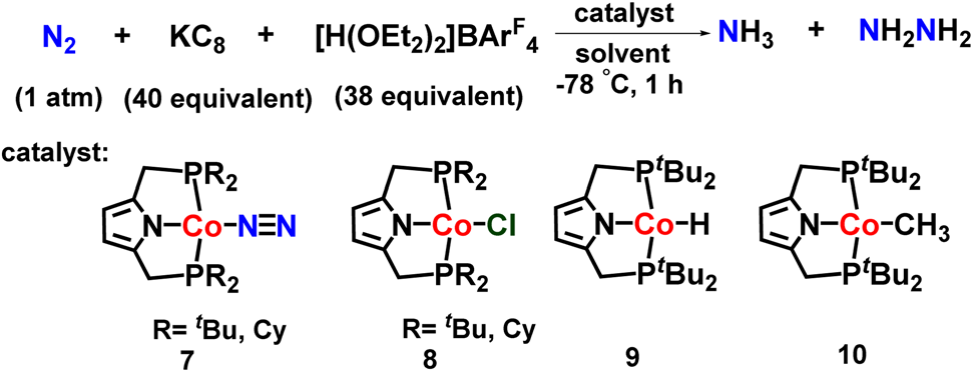
Cobalt complexes towards ammonia formation.

The same group also investigated other transition metal complexes as catalysts for nitrogen fixation. They reported a cobalt dinitrogen complex consisting of an anionic pincer-type PNP ligand for catalytic preparation of ammonia from dinitrogen (Fig. 12). The utilization of a bulk amount of reductant (KC_8_) and excess [H(OEt_2_)_2_]BAr^F^_4_ as a proton source gave the highest yield of ammonia (15.9 equiv.) and hydrazine (1.0 equiv.) based on Co catalyst.^47^

**Fig. 13:**
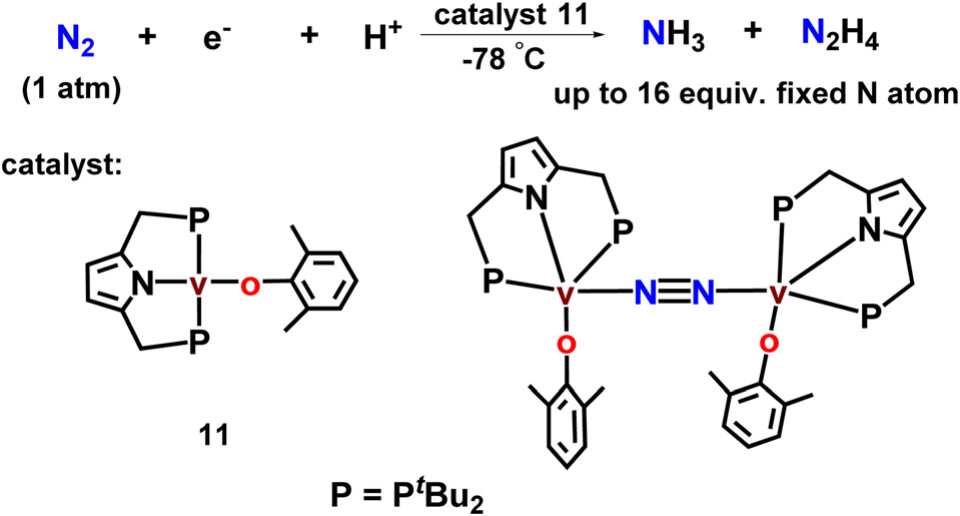
Pyrrole based vanadium complexes as catalysts toward nitrogen fixation.

Again, this group has newly designed and developed new vanadium complexes with an anionic pyrrole functionalized PNP-based pincer along with aryloxy ligands which worked as efficient catalysts towards the direct catalytic dinitrogen reduction to ammonia and also hydrazine at room temperature and pressure (Fig. 13). Up to 14 equiv. and 2 equiv. of ammonia and hydrazine (16 equiv. of fixed N atom) were produced respectively based on the vanadium atom. This was the first reported example of early transition metal-catalyzed reduction of dinitrogen under ambient reaction conditions.^48^

**Fig. 14:**
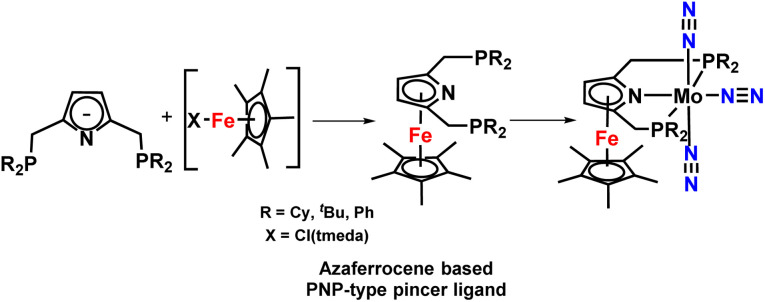
Azaferrocene-supported PNP pincer ligands for ammonia synthesis.

This group also investigated a set of azaferrocene-supported PNP-based pincer complexes of molybdenum, chromium, and iron complexes as catalysts for nitrogen fixation (Fig. 14). But unfortunately, these complexes were not very effective catalysts for catalytic alteration of dinitrogen to ammonia but proved to be efficient for catalytic silylamine formation from dinitrogen.^49^

**Fig. 15:**
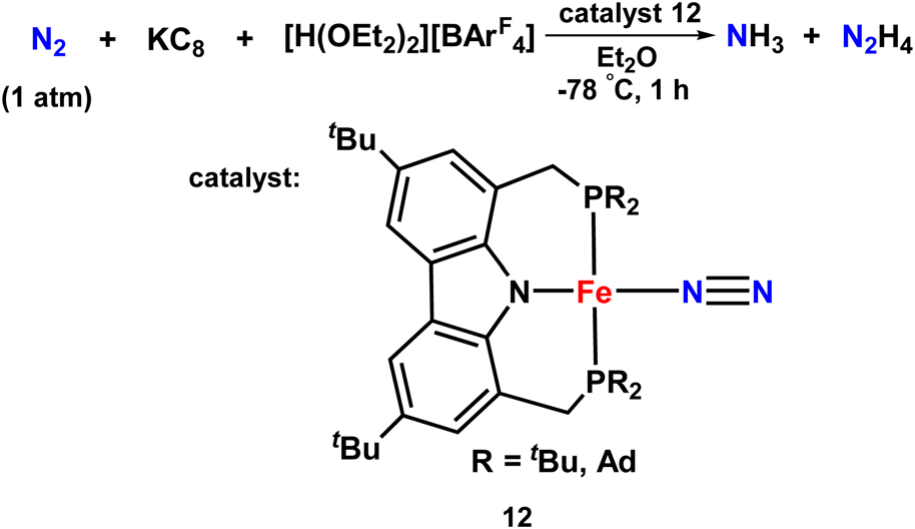
Iron complexes with a carb-PNP-based pincer ligand towards ammonia formation.

In 2017, an iron-dinitrogen catalyst possessing a dimethyl-substituted pyrrole bound PNP-type pincer ligand was explored, which functioned as an effective and better catalyst compared to that of an unsubstituted pyrrole bound PNP-type ligand towards the catalytic formation of ammonia plus hydrazine under an encompassing atmosphere. 22.7 and 1.7 equiv. of NH_3_ and N_2_H_4_ respectively, based on iron atom were obtained.^50^ Nishibayashi group also designed and synthesized new Fe–dinitrogen complexes consisting of an anionic carbazole functionalized PNP-based pincer ligands, [Fe(N_2_)(carb-PNP)] where carb-PNP = 1,8 bis(dialkylphosphinomethyl)-3,6-di-*tert*-butyl-carbazolide, and explored its catalytic activity in nitrogen fixation reaction (Fig. 15). The prepared iron complexes consisting of a carb-PNP-type pincer ligand provide a structure which is tetrahedral in geometry surrounding the Fe atom, and they claimed that the molecular structure of the iron complex had a significant influence on its catalytic activity in the complex.^51^

**Fig. 16:**
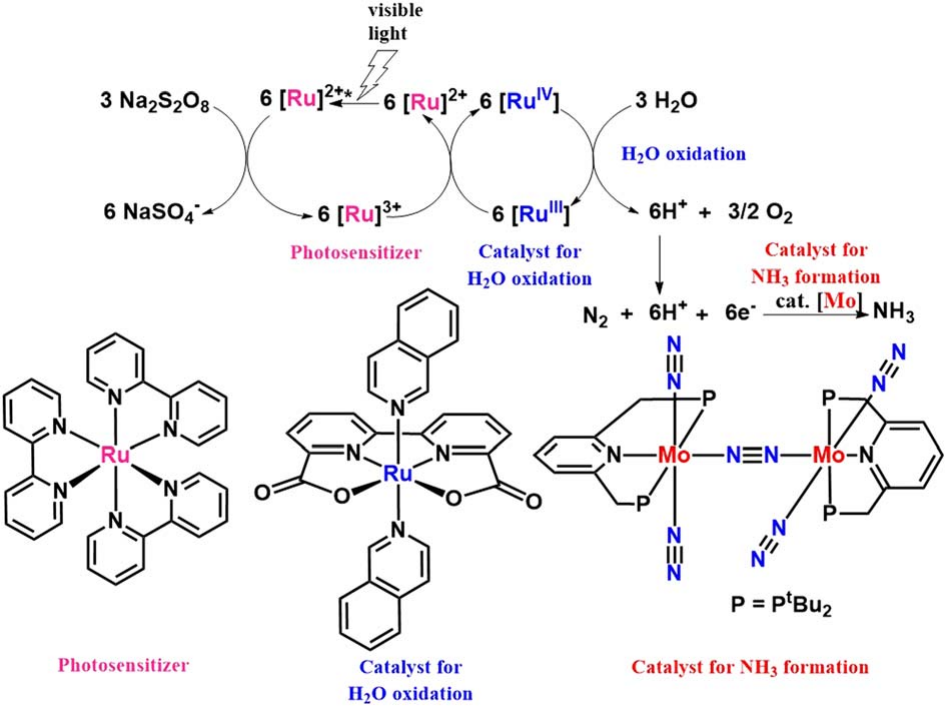
Visible light assisted Mo catalyzed dinitrogen transformation into ammonia employing a ruthenium water oxidation catalyst in the presence of a photosensitizer.

Nishibayashi group in 2017 also reported molybdenum catalyst to directly convert dinitrogen to ammonia employing a proton source formed *in situ* in the oxidation of water catalyzed by ruthenium catalyst in visible light in the presence of a photosensitizer (Fig. 16). The reaction system was considered a new model system for fixing dinitrogen by photosynthetic bacteria.^52^

This group put in another effort to bring light to the current scenario that the introduction of NHC-based ligands along with phosphine ligands could be beneficial for catalytically reducing nitrogen triple bond into ammonia. Therefore, a molybdenum catalyst bearing an NHC carbene alongside a phosphine-based pincer ligand, [{Mo(N_2_)_2_(PCP)}_2_(μ-N_2_)] was synthesized, which produced 230 equiv. ammonia as per the catalyst. According to theoretical studies, PCP ligand acts as a strong sigma donor as well as a pi acceptor, which leads to a strong bonding with the metal atom, thus improving the catalytic activity of the complex towards nitrogen fixation.^53^

**Fig. 17:**
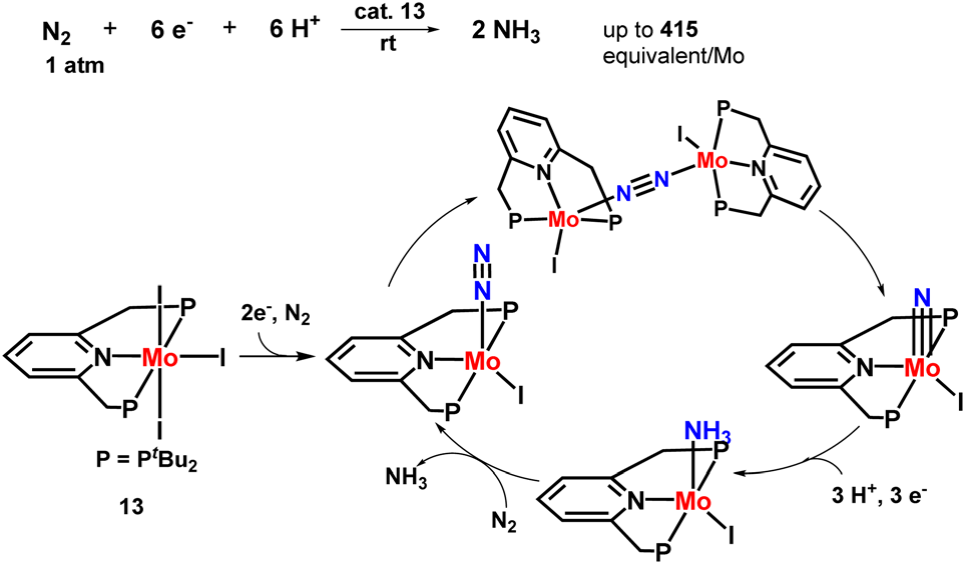
Mo–iodide complexes consisting of a PNP-based pincer ligand in ammonia formation.

Nishibayashi and co-workers then explored the catalytic preparation of ammonia in a slightly different approach. Different PNP-based pincer molybdenum–iodide complexes were prepared that showed higher catalytic activity towards ammonia synthesis than the molybdenum–dinitrogen catalysts reported till date at ambient conditions (Fig. 17). 830 equiv. of ammonia were obtained based on the complex (415 equiv. ammonia achieved based upon one molybdenum atom). Direct cleavage of nitrogen–nitrogen triple bond is promoted by the generation of dinitrogen-bridge between two molybdenum–iodide complexes, which is supposed to be an essential point in this novel reaction pathway. To understand this remarkable catalytic activity, further research was carried out which was necessary to interpret the mechanistic pathway in the reaction.^54^

**Fig. 18:**
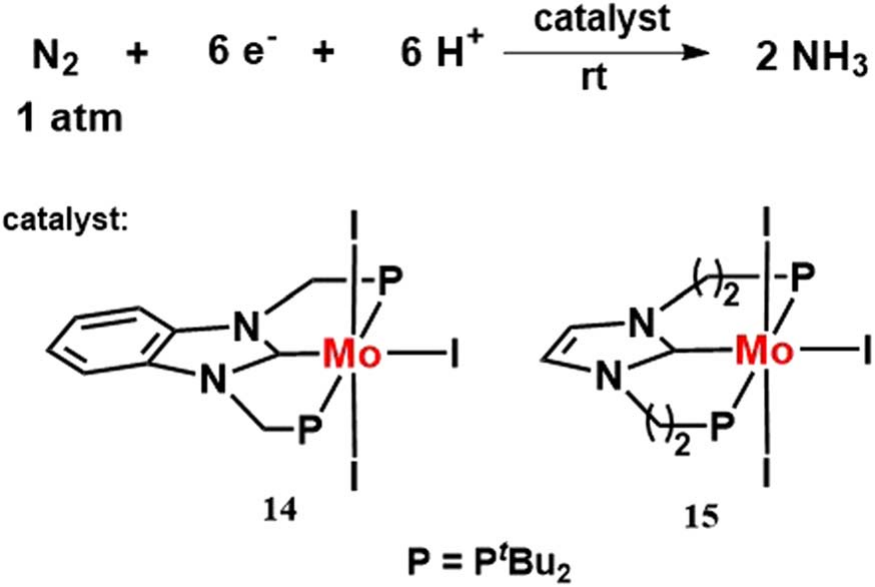
Molybdenum iodide complexes bearing PCP ligand towards nitrogen fixation.

In 2019, two different type of molybdenum triiodide complexes having a PCP ligand, [MoI_3_(PCP)] were prepared (Fig. 18). Its catalytic activity towards nitrogen fixation was explored in comparison to the previously prepared [{Mo(N_2_)_2_(PCP)}_2_(μ-N_2_)], [MoI_3_(PNP)] complexes. [MoI_3_(PCP)] complex was found to work best as a catalyst among the four molybdenum complexes in producing ammonia under identical reaction conditions.^55^

**Fig. 19:**
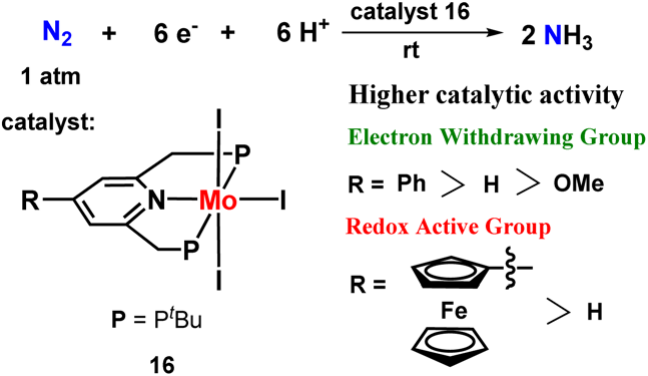
Substituted pyridine-based molybdenum triiodide pincer complexes towards nitrogen fixation.

Next, they envisaged a group of molybdenum triiodide catalysts to fix dinitrogen into ammonia at room temperature. Different substituents in the pyridine ring of the PNP ligand were synthesized and screened. The introduction of different substituents like electron withdrawing phenyl group and redox-active ferrocenyl group to the 4^th^ place of pyridine ring resulted in a substantial increment of the catalytic activity for the catalyst towards ammonia formation (Fig. 19).^56^

**Fig. 20:**
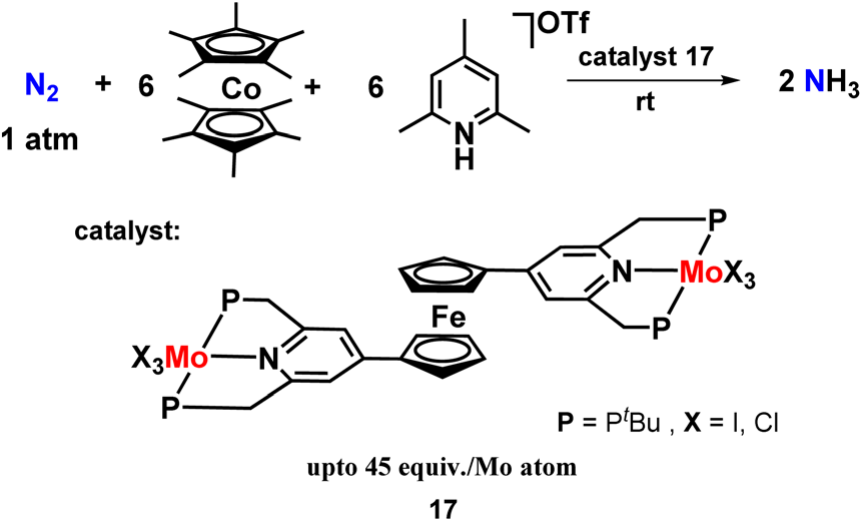
Ferrocene co-ordinated bis(molybdenum-trihalide) complexes towards ammonia synthesis.

Keeping this in mind, they designed and prepared bis(molybdenum-trihalide) PNP-based pincer complex linked by a ferrocene skeleton and explored its catalytic activity in the synthesis of ammonia (Fig. 20). But unfortunately, they found that the ferrocene-bridged dimolybdenum complex shows poor catalytic activity compared to the mononuclear Mo-trihalide complexes under similar reaction conditions. From the DFT study, the intramolecular bridge formation of dinitrogen between the two molybdenum atoms required for N_2_ cleavage was not observed in the ferrocene-linked dimolybdenum complexes. They claimed that this was because of the steric hindrance induced by the tertbutyl groups on the phosphorus atoms, which inhibited the bridge formation. Thus, they envisaged the unsuitable design of the catalyst for intramolecular N_2_ bond cleavage to be the sole reason for less catalytic activity in ammonia production.^57^

**Fig. 21:**
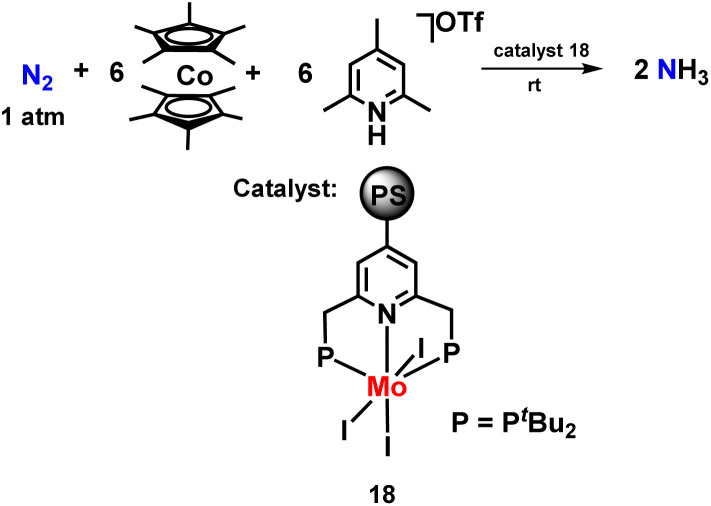
Polystyrene-assisted molybdenum trihalide catalyst with a pyridine-based PNP pincer ligand for ammonia preparation.

Nishibayashi and co-workers then synthesized a polystyrene-assisted molybdenum trihalide catalyst with pyridine-based pincer ligand to investigate their catalytic reactivity towards the reduction of molecular dinitrogen to ammonia at mild reaction conditions (Fig. 21). Decamethylcobaltocene was used as a reductant, and 2,4,6-collidinium trifluoromethanesulfonate was used as a proton source along with the catalyst. They found that these complexes worked as suitable heterogeneous catalysts for ammonia formation at room temperature.^58^

**Fig. 22:**
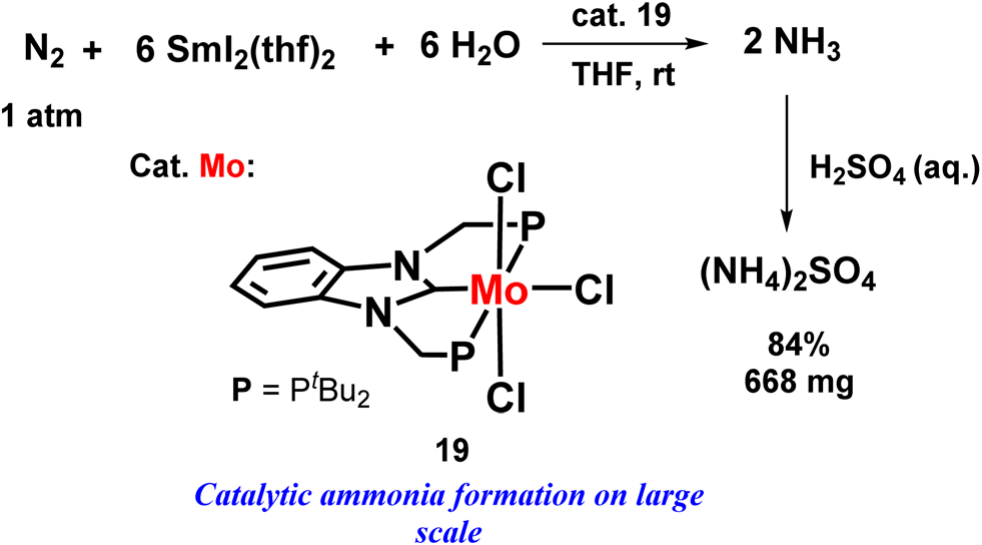
Molybdenum-catalysed nitrogen fixation using samarium diiodide with water.

An eye-catching or marvellous success was achieved by the Nishibayashi group for catalytic ammonia preparation from dinitrogen when molybdenum catalyst was coupled with SmI_2_ and water or alcohol. This was another new approach of this group where they mainly emphasized the reaction conditions where they used a mild reducing agent and a low-cost or cheap proton source, which led to the high yield of ammonia using different types of catalysts. The amount of ammonia obtained was 10 times higher than in the previously reported reaction systems. The molybdenum trichloride complex with PCP ligand showed much higher catalytic activity both in the case when ethylene glycol (3650 equiv. ammonia was achieved from one molybdenum atom; 76% yield obtained on SmI_2_) and water (4350 equiv. ammonia was achieved from one molybdenum atom; 91% yield obtained on SmI_2_) was used separately than the molybdenum triiodide catalyst possessing a PNP type ligand. They investigated this reaction condition on a larger scale, and found that the catalytic reaction occurred quite rapidly, in 3 minutes, also on a higher scale. (NH_4_)_2_SO_4_ Could be isolated in more than 500 mg from the accomplished reaction mixture (Fig. 22).^59,60^

**Fig. 23:**
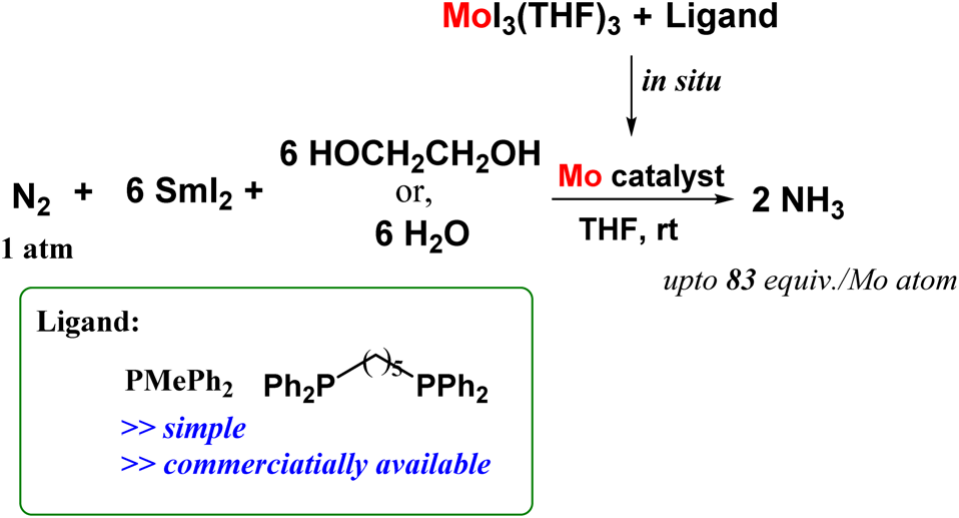
Ammonia production at ambient reaction conditions by molybdenum complexes formed *in situ* by reacting [MoI_3_(THF)_3_] and monodentate and bidentate phosphines.

After an extensive study in this field, they came up with a novel reaction system that in a simple and convenient route, could successfully lead to ammonia production under ambient reaction conditions. They reported molybdenum complexes induced from [MoI_3_(THF)_3_] and auxiliary ligands such as bidentate phosphines like 1,5-bis(diphenylphosphino)pentane and monodentate phosphines like PMePh_2_ which worked productively to give ammonia (Fig. 23). The reaction of [MoI_3_(THF)_3_] and auxiliary ligand in the presence of N_2_ atmosphere was carried out with SmI_2_ and ethylene glycol/water since this combination of reductant and proton source respectively proved to be an efficient reaction system towards ammonia formation as previously stated. In the present case, when the reaction was done using [MoI_3_(THF)_3_] and dppe, using higher amounts of water and SmI_2_, the amount of ammonia obtained was 83 equiv. based on the Mo atom.^61^

**Fig. 24:**
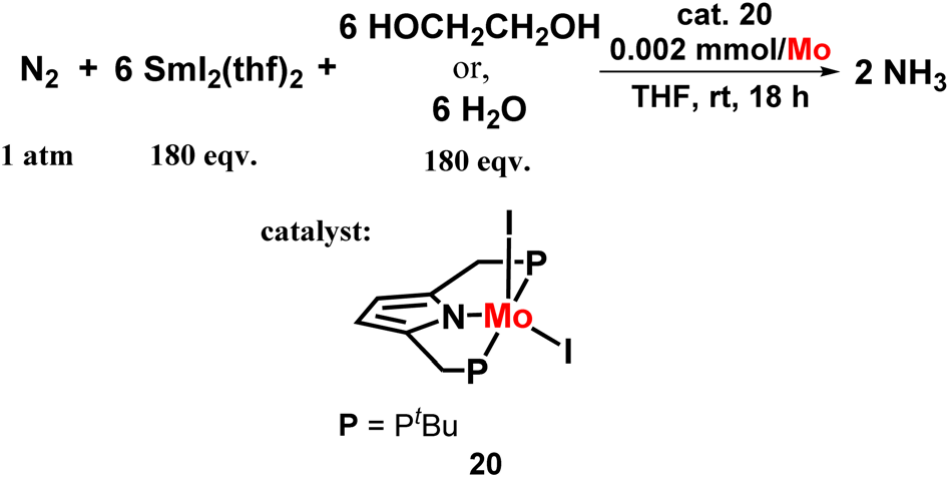
NH_3_ formation by molybdenum complex consisting of a PNP type anionic pyrrole ligand.

In 2020, Nishibayashi and his co-workers published another work that showed the synthesis of molybdenum complexes bearing a PNP type pyrrole-based anionic pincer ligand where they found these catalysts to be effective towards NH_3_ formation under favourable reaction conditions (Fig. 24). Nitride species is formed as a prior intermediate *via* the N≡N triple bond cleavage, which facilitates ammonia formation.^62^

**Fig. 25:**
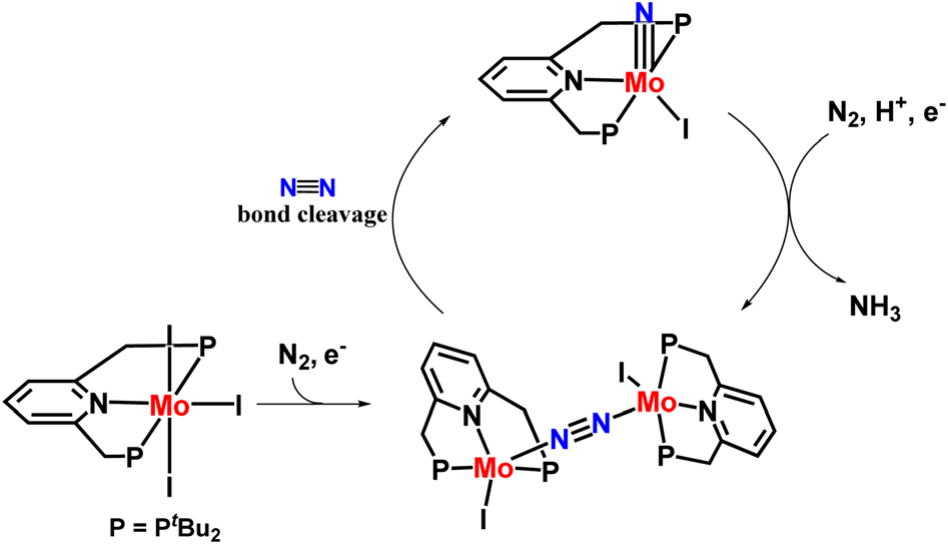
Molybdenum dinitrogen complex as an important species in the nitrogen fixation cycle.

Dispersive XAFS spectroscopy technique was used to characterize the molybdenum–dinitrogen complex, which was presumed to be a key species in the stoichiometric conversion of molybdenum triiodide [MoI_3_(PNP)] complex into molybdenum nitride [(Mo≡N)(PNP)I] complex under an atmospheric pressure of dinitrogen (Fig. 25).^63^

**Fig. 26:**
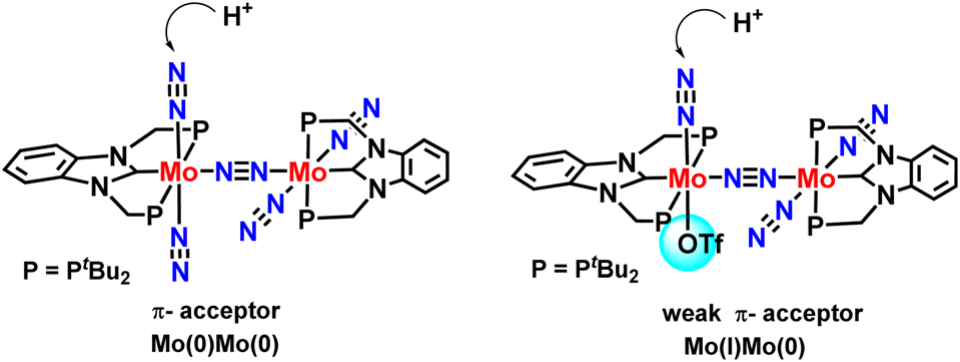
NHC-based N_2_ bridged dimolybdenum catalysts for nitrogen fixation.

Density functional theories by this group suggested a new plausible mechanism for nitrogen fixation for complexes containing PCP & PNP pincer-type ligands. According to DFT calculations, a new mechanism could be proposed based on which it could be inferred that dinuclear Mo–N≡N–Mo structural binding should be preserved during the catalytic cycle. Also, they found that dinitrogen co-ordinated at Mo(i) center bound to the electron-releasing triflate group shows higher reactivity towards protonation than the Mo(0) center (Fig. 26).^64^

In 2021, Nishibayashi *et al.* developed an electrochemical pathway for ammonia synthesis using SmI_2_ as a reductant which was prepared from SmI_3_ along with H_2_O as a proton source. Here, electrochemical energy was transformed into chemical energy by the formation of ammonia using samarium iodide with high faradaic efficiency.^65^

**Fig. 27:**
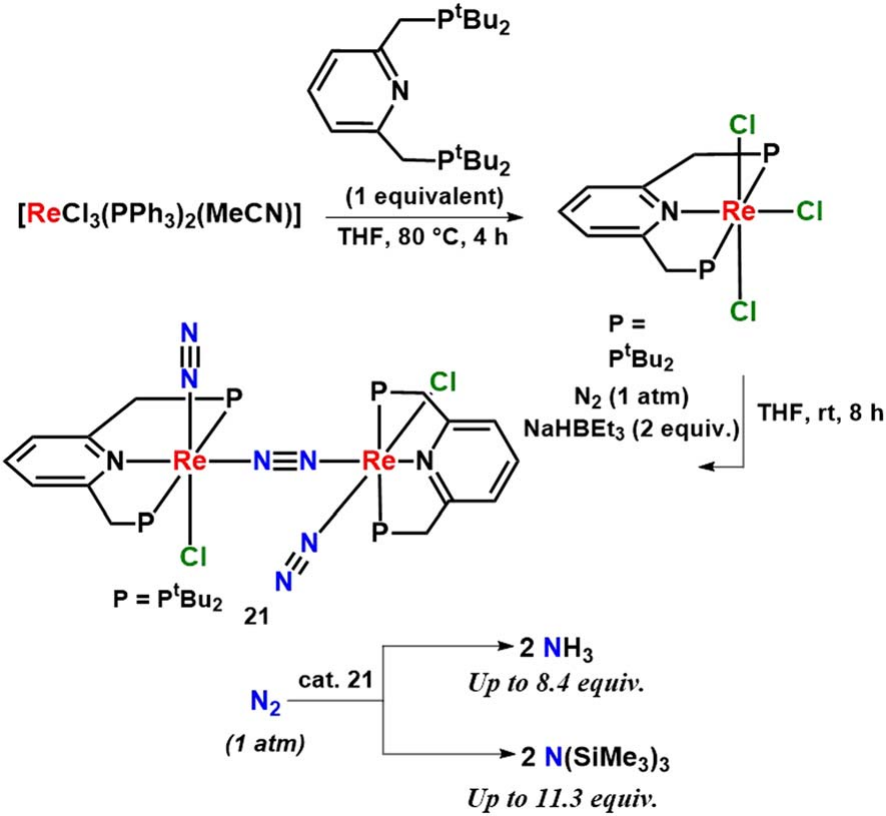
A dirhenium catalyst with a dinitrogen bridge for ammonia and silylamine preparation.

Different rhenium complexes having pyridine ligands were developed which catalyzed dinitrogen conversion into ammonia at room temperature and pressure. This was the first reported example of a rhenium dinitrogen complex, which can catalyze nitrogen fixation at room temperature. 8.4 equiv. of ammonia was obtained as per the catalyst (Fig. 27).^66^

**Fig. 28:**
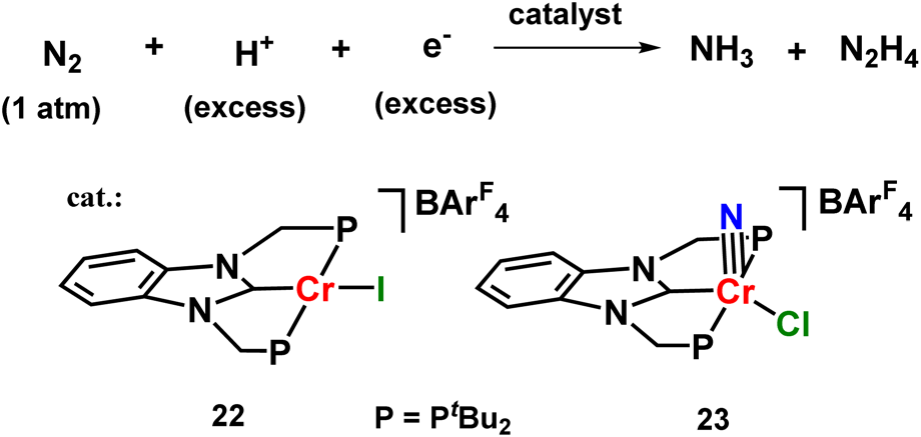
Chromium complexes towards catalytic N_2_ fixation.

Similar results were also observed for chromium halide catalysts based on PCP-type of pincer ligand which produced ammonia as well as hydrazine at atmospheric pressure and room temperature in 8.4 equiv. and 2.46 equiv. yields respectively (Fig. 28).^67^

**Fig. 29:**
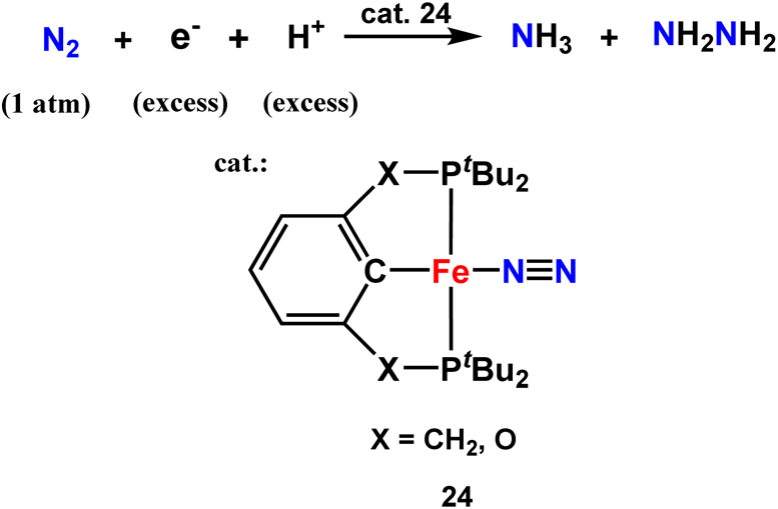
Iron dinitrogen complex as a catalyst for ammonia formation.

Recently, iron dinitrogen complexes were synthesized by this group to explore their activity towards nitrogen fixation to get ammonia under mild conditions (Fig. 29). The ligand system was a benzene co-ordinated PCP- and a POCOP-based pincer which binds to the metal center to catalytically fix dinitrogen into ammonia and hydrazine with 252 equiv. and 68 equiv. (388 equiv. N atom was fixed) based on the iron atom. Among the reported iron catalysts, the iron(i)-PCP catalyst produces the highest quantity of ammonia and hydrazine till date.^68^

Most recently, three different manganese complexes were also explored by Nishibayashi *et al.* towards nitrogen fixation into ammonia and silylamine formation, but unfortunately, only stoichiometric amount could be achieved. The reason that could be inferred was the unstable nature of the synthesized manganese pincer complexes under reducing conditions which ultimately causes catalyst decomposition.^69^

### 3.3. J. C. Peter's work

J. C Peters and his co-workers in 2013 described tris(phosphine)borane-assisted iron complex, which helps produce NH_3_ at ambient conditions. In this work, more than 40% of the proton equiv. as well as reducing equiv. are supplied to N_2_.^26^

This group also extended their work to synthesize Fe(i) complexes bearing tris(phosphine)borane ligand featuring hydrazine, ammonia, amine, and hydroxy groups as ligands. Reductive substitute to NH_2_ group in the terminal position in the Fe–NH_2_ species by N_2_, along with simultaneous liberation of NH_3_ leads reliance to a certain pathway which is mechanistically feasible with Fe–conciliated dinitrogen reduction schemes.^70^

**Fig. 30:**
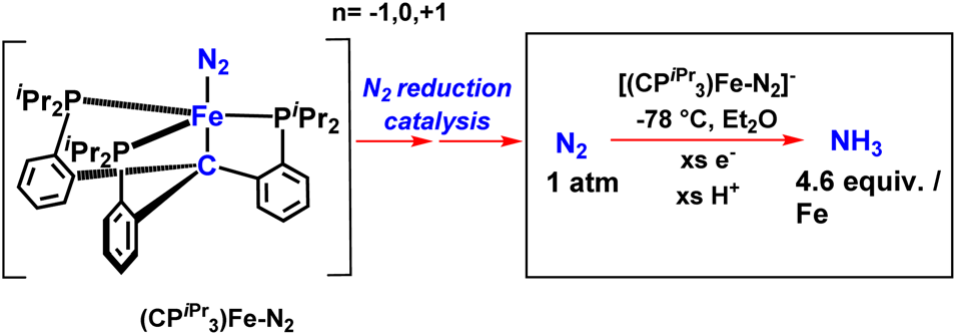
Ammonia formation using a Fe–N_2_ catalyst showing C atom as an anchor.

Again in 2013, they published work on iron complexes bearing a novel tris(phosphino)alkyl (CP^*i*^Pr_3_) ligand highlighting the axial carbon donor wherein they hypothesized the idea of trans interaction of the C-atom with an iron center to reveal the Fe–N_2_ binding position (Fig. 30). In this arena, the iron center holds dinitrogen and the C_alkyl_-atom anchor trans to each other in three well defined and structurally distinguished oxidation states. Upon reduction, Fe–C_alkyl_ bond lengthening was observed, which signifies the presence of ionic nature in the Fe–C_alkyl_ interplay. When provided with protons and electrons at −78 °C, with nitrogen atmosphere, 4.6 equiv. NH_3_ per Fe atom was produced. (CP^*i*^Pr_3_)FeN_2_^−^ operates as a decent catalyst for nitrogen triple bond reduction to ammonia.^71^

In the same year, Peter's group also reported a string of four- & five-coordinated iron complexes that display an axially positioned tri(silyl)methyl ligand oriented trans to that of a substrate holding site. However, this disposition was to solely depict a single site of the Fe atom of the so-called FeMo-cofactor, which perhaps binds N_2_*trans* to the C atom at the interstitial site. The data obtained were placed in context, supporting a hypothesis towards substrate binding and reduction, which is facilitated by the controlling influence of belt Fe–C interplay in FeMo-cofactor.^72^

**Fig. 31:**
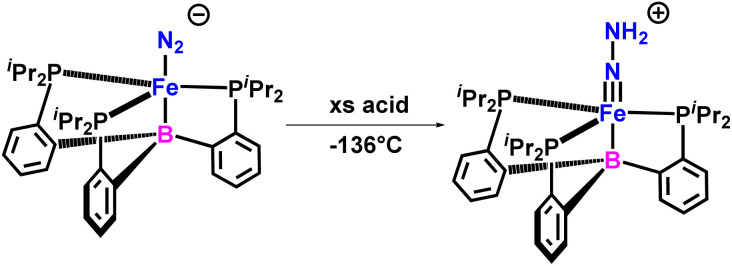
Fe≡N–NH_2_ Intermediate for catalytic Reduction of N_2_.

In 2015, they characterized Fe≡N–NH_2_ intermediate formed by directly protonating Fe–N_2_ species pertinent to the catalytic reduction of N_2_ (Fig. 31). The newly formed species as characterized was a hydrazido(2^−^) complex, [(TPB)Fe≡N–NH_2_]^+^ (where TPB stands for tris(phosphine)borane), which is a doubly protonated unstable species having a Fe≡N bond. It offers a solid argument establishing the fact that the initial steps during the Fe-mediated reduction of dinitrogen by [(TPB)Fe(N_2_)][Na(12-crown-4)] may progress by a distal or follow a “Chatt-type” reaction pathway.^73^

Another work of the Peters group was the preparation of a five-coordinated diamagnetic species Fe=NNH_2_^+^, assisted with a tris(phosphino)silyl ligand and isolation of the same. This species was obtained *via* direct protonation from a Fe–N_2_^−^ complex, which was terminally bound. It was found that Fe=NNH_2_ expeditiously transforms into Fe–NH_2_NH_2_^+^ species at warmer temperatures *via* an additional conveyance of proton and electrons in solution. It was observed that Fe–NH_2_NH_2_^+^ could liberate NH_3_. Thus, it was evident from the series of reactions reported herein that a Fe site can move from a distant intermediate (Fe=NNH_2_^+^) to a proximal intermediate (Fe–NH_2_NH_2_^+^) which is in transit to NH_3_ generation from N_2_.^74^

**Fig. 32:**
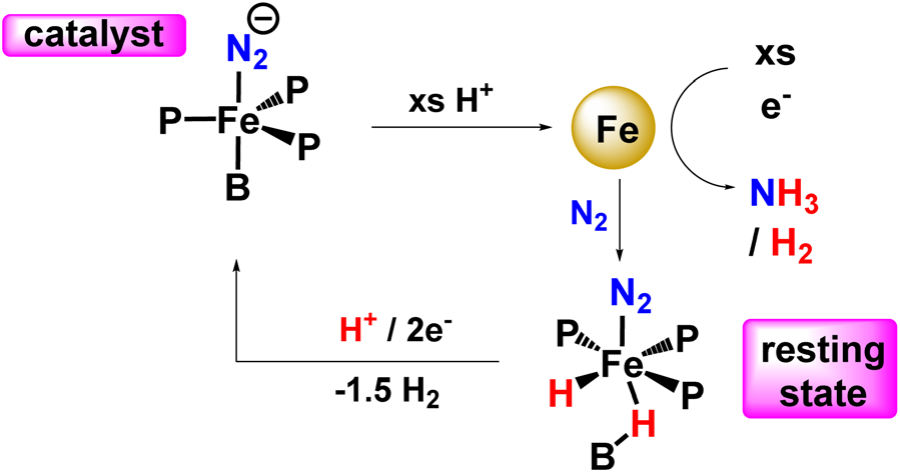
A synthetic strategy for a single-site iron nitrogenase towards ammonia formation.

In 2016, Peters and his co-workers developed dinitrogen fixation catalyst systems with the help of P_3_^E^Fe (E refers to B/C/Si) species that gave rise to an elevated amount of ammonia if adequate acid (proton source) and reductant were supplied (Fig. 32). They found these iron catalysts to be fortuitously robust and endure its activity after a number of reloadings as well. According to Mössbauer spectroscopy, it can be revealed that during the turnover of the P_3_^B^Fe catalyst system, a Fe-borohydrido-hydride species appears to be likely in a resting state. Therefore, they postulated that the evolution of hydrogen reaction activity might inhibit poisoning of the P_3_^B^Fe system by preventing the formation of iron hydride species. This proposition was fundamental to account for the synthetic scheme and design of nitrogenases, and it might also have a great significance as can be known that evolution of hydrogen and metal hydride intermediates may play a crucial role in naturally fixed N_2_ into ammonia.^75^

Another new strategy was employed by this group to liberate ammonia and methane with the help of proton and electron equivalents by using an iron cyanide complex, [SiP^*i*^Pr_3_]Fe(CN) as a catalyst (where, [SiP^*i*^Pr_3_] = tris(phosphine)-silyl ligand). According to preliminary mechanistic studies, [SiP^*i*^Pr_3_]Fe(CN) additionally served as a handy door opener to limited examples having terminally bound species like Fe(CNH) and Fe(CNH_2_), which can be probable intermediates of reductive protonation of cyanide to methane and ammonia.^76^

**Fig. 33:**
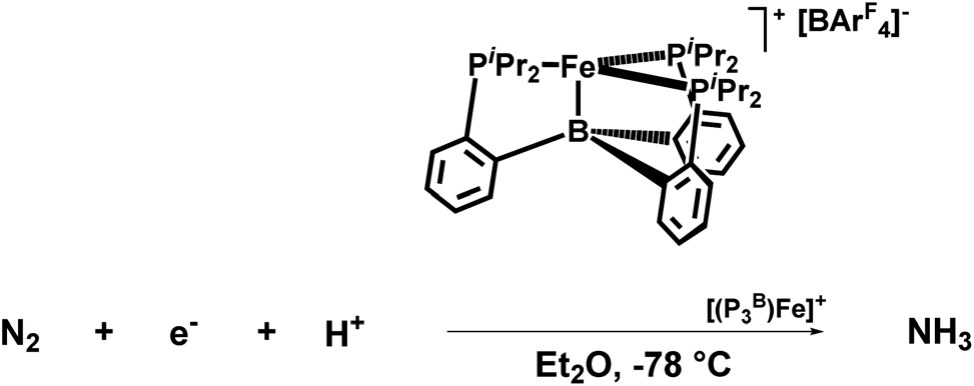
Catalytic conversion of N_2_ to NH_3_ by P_3_^B^Fe^+^ catalyst.

Peters group explored a Fe catalyst that functions at −78 °C and also at atmospheric pressure for the alteration of N_2_ to ammonia (Fig. 33). KC_8_ was used as a potent reducing agent, and [H(OEt_2_)_2_][BAr^F^_4_] was used as the acid source. Their catalyst P_3_^B^Fe^+^ (P_3_^B^ = tris(*o*-diisopropylphosphinophenyl)-borane) system exhibits both significantly enhanced efficiency for NH_3_ generation (up to 72% of e^−^ supply) as well as a relatively high turnover number to a molecular iron catalyst. They achieved up to 84 equiv. NH_3_ for each Fe site), while using a specifically weaker amalgamation of reducing agent 
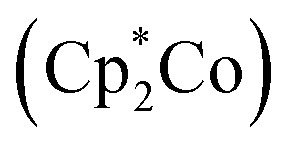
 and proton source ([Ph_2_NH_2_][OTf] or [PhNH_3_][OTf]).^77^

**Fig. 34:**
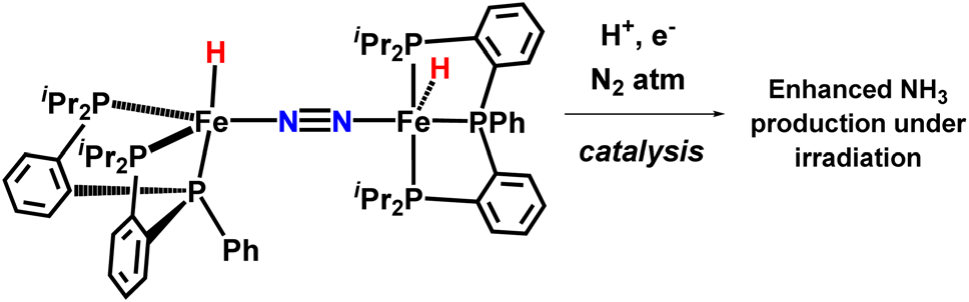
Light driven nitrogen fixation by triphos-supported Fe catalysts.

In 2017, the Peters group synthesized and characterized new catalytic systems described as P_2_^P’Ph^Fe(N_2_)(H)_*x*_, which are effective for catalytic NH_3_ synthesis (Fig. 34). It was found that if catalysis were to be carried out in the presence of a mercury lamp irradiation, the ammonia production yield was significantly increased. Evidence was provided to suggest that this enhanced activity may arise due to photo-elimination of H_2_.^78^

**Fig. 35:**
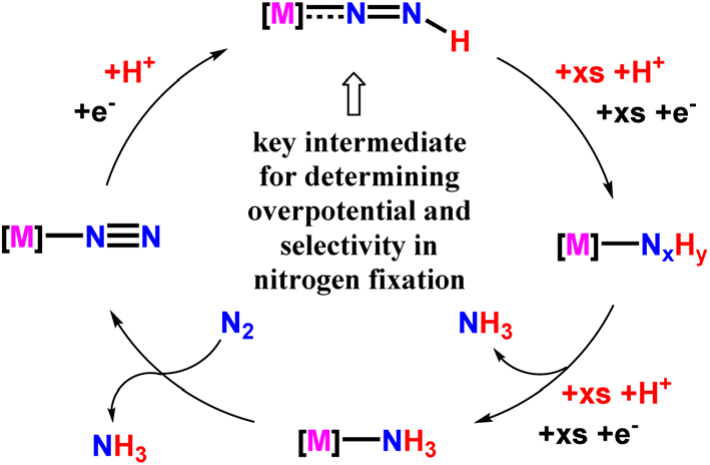
Transition metal mediated catalytic N_2_RR mechanism for ammonia synthesis.

Again, another approach to synthesizing ammonia by this group was to investigate the relation between the bond dissociation free energy (BDFE) of N–H bond of a M-NNH species with observed over potential alongside selectivity for N_2_RR (nitrogen reduction reaction) catalysis for that very catalyst system (Fig. 35). They emphasized that the development of different approaches or strategies to help increase the BDFEs of N–H bond in a M-NNH species, or avoiding M-NNH intermediates as a whole, can be potential routes for improved N_2_RR efficiency.^79^

**Fig. 36:**
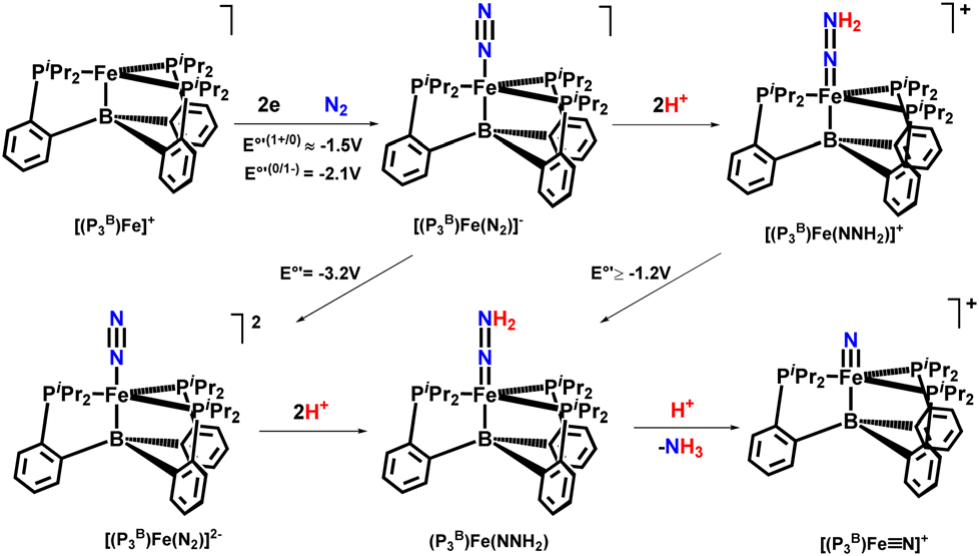
Nitrogen fixation *via* a terminal Fe(iv) nitride.

Then again in 2017, this group showed that on protonation of a Fe–N_2_ nitrogen-fixing catalyst results in forming a Fe(NNH_2_)hydrazido(2−) neutral intermediate. This intermediate species can undergo further protonation and release [Fe^IV^≡N]^+^ and NH_3_ by heterolytically cleaving the N–N bond. These observations provided by them depict direct affirmation of the feasibility of a mechanism similar to Chatt for iron-mediated dinitrogen change into ammonia (Fig. 36).^80^

In 2018, Peters and his co-workers depicted the first orderly p*K*_a_ studies about an artificial nitrogen fixation catalyst where they found a strong connection between the p*K*_a_ and N_2_RR efficacy *vs.* HER (hydrogen evolution reaction) efficacy. Chemical studies revealed that, in catalysis, anilinium triflate acids were unable to bring about the N–H bonds in initial stage intermediates of N_2_RR like P_3_BFeNNH_2_^+^. They considered that the reasonably rapid proton transfers essential to captivate the unstable critical first fixed intermediate, P_3_BFeNNH is prevented by the insoluble nature of the triflate acids. Under catalytic conditions, the presence of 
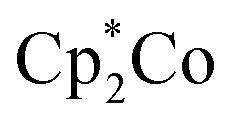
 as reductant is vital, as protonation can occur *in situ* with the formation of Cp*(η4-C_5_Me_5_H)Co^+^, that in a way is operative for the formation of N–H bond along with early intermediates.^81^

**Fig. 37:**
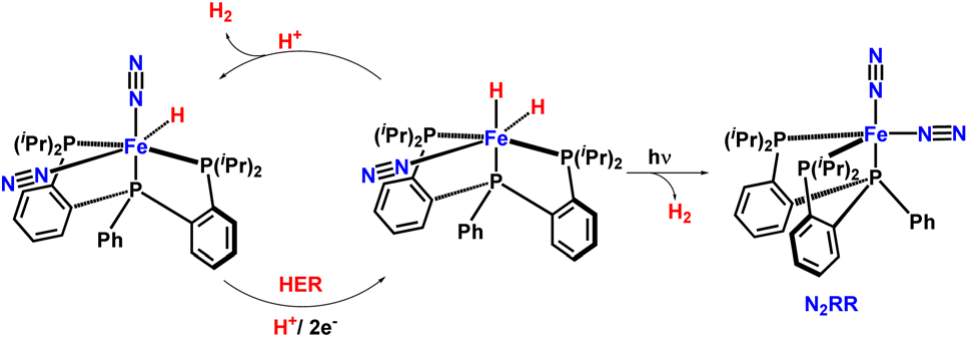
Mechanistic insights in H_2_ elimination, HER, and NH_3_ formation using Fe catalyst under irradiation of light.

In 2019 they highlighted the adverse impact of hydride ligands on a Fe-catalyzed N_2_RR (nitrogen reduction reaction) system wherein efficiency was enhanced by irradiation (Fig. 37). It was observed that in a Fe-based system having trisphosphine ligand, the presence of one, two, or zero hydride ligands in N_2_RR precatalysts results in dramatically better yields, although irradiation of the catalysts with the help of a mercury lamp ensues similar NH_3_ yields.^82^

Soon after, they also studied a series of redox innocent ligand Fe(NNR_2_)^+/0/−^ and their electronic properties. They envisaged that the redox noninnocence of the “NNR_2_” ligand might exhibit itself in various examples relating to transition-metal catalyzed N_2_ fixation.^83^

Peters *et al.* in 2021 developed iron-dinitrogen complexes consisting of tris(phosphino)alane (P_3_Al) as well as tris(phosphino)gallane (P_3_Ga) ligands to check their nitrogen reduction reaction (N_2_RR) activity in nitrogen fixation. They envisioned these complexes to have similar N_2_RR activity to those of earlier reported tris(phosphino)borane (P_3_B) type Fe–N_2_ complex due to their similar electronic structures, geometric flexibility in Fe→X (X = Al, Ga) interactions, and degree of N_2_ activation as obtained from spectroscopic, electrochemical, structural, and DFT studies. But on the contrary, ammonia yield was reduced with the use of an excess amount of acid HBAr^F^_4_ and reducdant KC_8_. Relative to the P_3_BFe system, the attenuated yield of ammonia with P_3_AlFe and P_3_GaFe dinitrogen complexes was explained to be due to their greater hydrogen evolution reaction (HER) selectivity than N_2_RR as could be presumed from their reactivity studies which suggest robustness of these catalysts in pseudo catalytic conditions.^84^

**Fig. 38:**
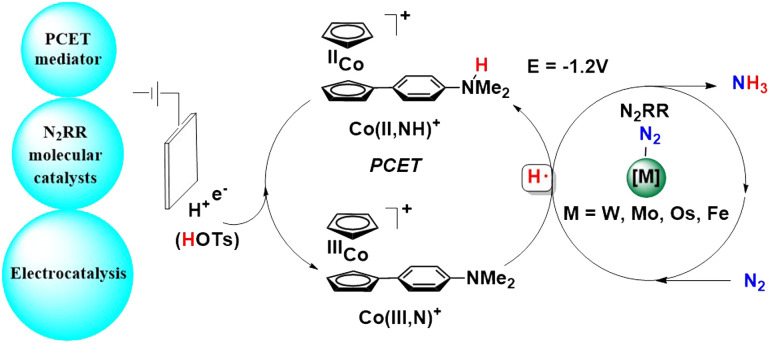
Tandem approach to electro-catalytically convert dinitrogen into ammonia.

With this result in mind, they designed a tandem approach to electro-catalytically facilitate dinitrogen reduction into ammonia (Fig. 38).^85^ Here, a co-catalyst was used along with a molecular complex that interfaces both the electrode as well as the acid so as to mediate the N_2_ reduction cycle by facilitating the CPET (concerted proton–electron transfer) steps. This strategy enables the formation of an N–H bond at an applied potential that is promising and with high overall thermodynamic efficiency. This tandem catalysis was performed with complexes of W, Mo, Os, and Fe along with the CPET mediator, Co(II, NH)^+^ to achieve nitrogen reduction reaction (N_2_RR) electrocatalysis at mild potentials (−1.2 V using TsOH).

**Fig. 39:**
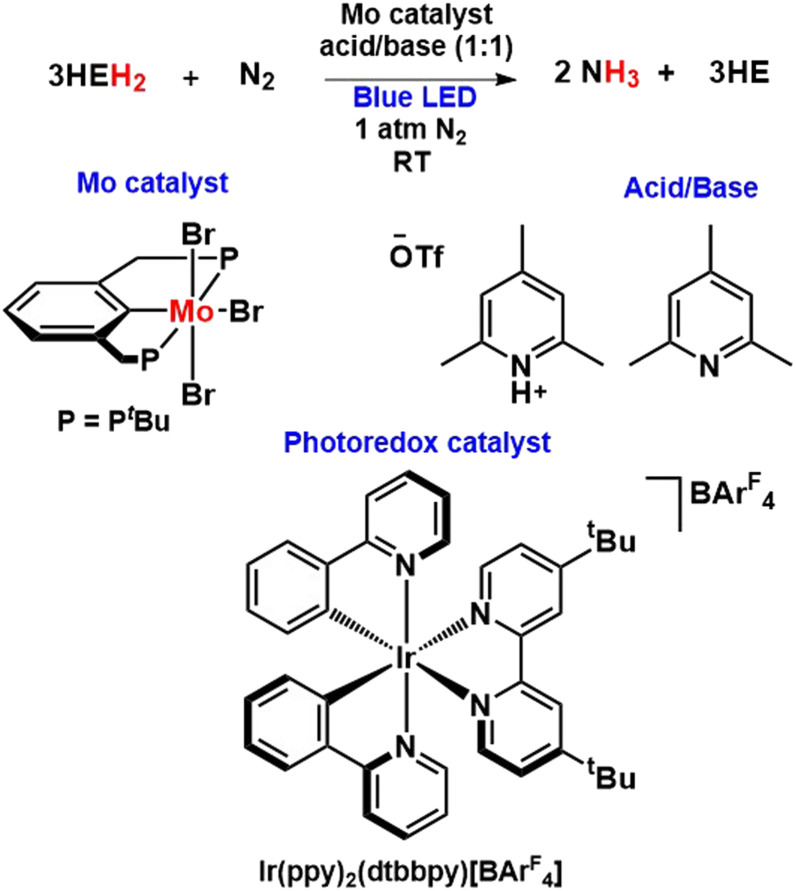
Photochemically induced transfer hydrogenation strategy for nitrogen fixation.

Most recently, Peters and his co-workers envisaged a photochemically induced transfer hydrogenation strategy for nitrogen fixation using a Mo catalyst with the help of Hantzsch esters (HEH_2_) as photoreductants (Fig. 39). In their work, they demonstrated the successive supply of H_2_ equivalents stored by completely reduced Hantzsch esters to N_2_ to catalytically produce NH_3_ in the presence of Mo pre-catalyst under irradiation of blue light. Besides, the addition of iridium photoredox catalyst, which is not mandatory for this photocatalysis to occur, increases the reaction rates as well as the turnover numbers of the net reaction.^86^

### 3.4. P. J. Chirik work

P. J. Chirik in 2015, synthesized a complex (η^5^-C_5_Me_4_SiMe_3_)_2_Ti(Cl)NH_2_ and carried out hydrogenolysis of Ti–NH_2_ bond to produce ammonia using Proton Coupled Electron Transfer (PCET) process.^87^ With the help of an appropriate Rh hydride catalyst, the H atom source used in stoichiometric amounts was from hydrogen to liberate ammonia out of coordination sphere around the Ti metal center.

**Fig. 40:**
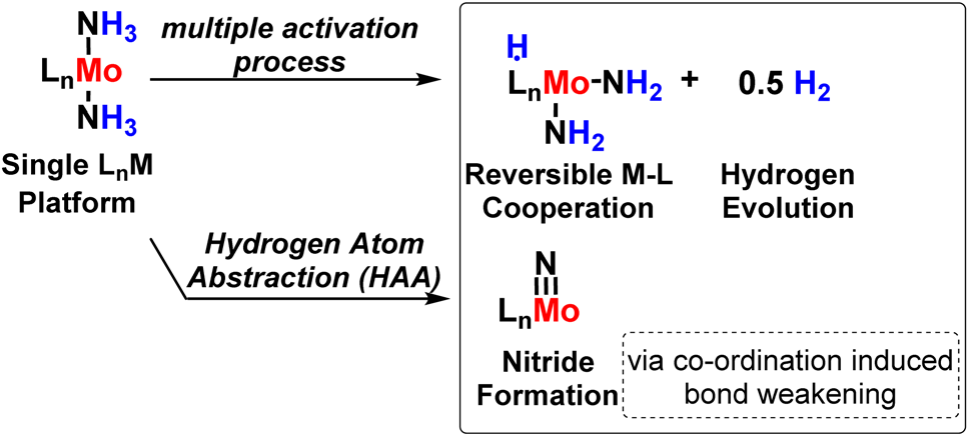
Strategy for ammonia activation.

A series of bis(cyclopentadienyl) Ti and Zr amide, hydrazide as well as imide complexes were explored by Chirik and his co-workers^88^ in 2016 for the development of the N–H bond, and the synthesis was based on the PCET mechanism based on the presence of stoichiometric H atom source as H_2_. Again, the Chirik group in 2017 developed a new molybdenum complex bearing a redox-active ligand which is a bis(imino)-pyridine ligand for N_2_ activation as well as H_2_ evolution and nitride formation (Fig. 40).^89^

**Fig. 41:**
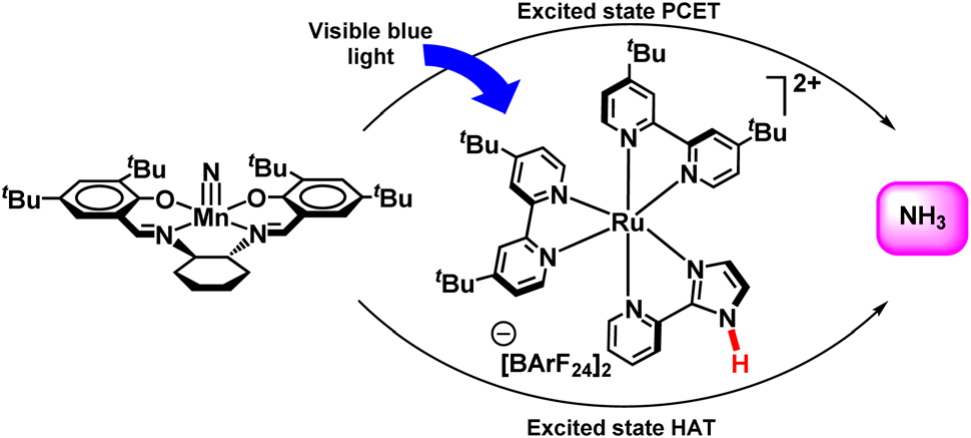
Ammonia formation from a manganese nitride using a Ru photocatalyst.

Soon after, in 2019, this group explored a different method to synthesize ammonia from a Mn nitride complex photochemically (Fig. 41). 9,10-Dihydroacridine was used along with a manganese nitride complex, (^*t*^BuSalen)MnN (^*t*^BuSalen = (*S*,*S*)-(+)-*N*,*N*′-Bis(3,5-di-*tert*-butylsalicylidene)-1,2-cyclohexanediamine) and a ruthenium photocatalyst in isopropanol solution which when exposed to radiation with blue light produced free ammonia. Besides the ground-state phenomenon, *i.e.* co-ordination induced weakening of bond; the extended concept of excited state bond dissociation free energy (BDFEs) of N–H was explored. On the other hand, photo-induced ammonia formation from manganese nitride complex with ruthenium complexes having distant N–H bonds has been potent, and control experiments showed that these ruthenium complexes behave more like photoreductant than hydrogen atom transfer (HAT) agents. Luminescence quenching studies suggested ruthenium(ii)/(iii) cycle rather than an alternative, ruthenium(i)/(ii). This supports the fact that electron as well as proton transfer takes place at different sites and thus showed a tentative PCET pathway in the excited state rather than hydrogen atom transfer.^90^

**Fig. 42:**
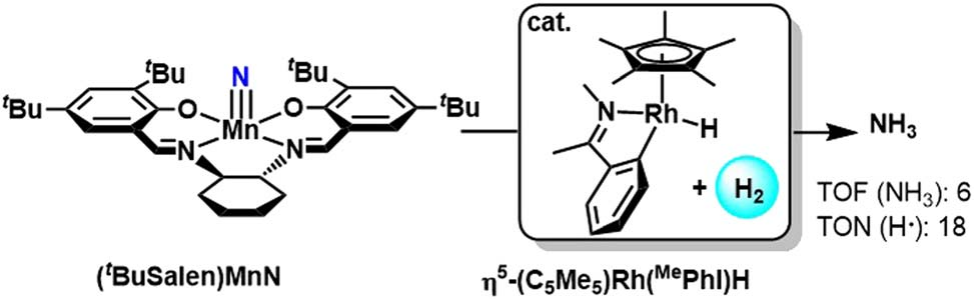
Hydrogenation of Mn(v) nitride complex for ammonia production.

Chirik group in 2020 showcased manganese(v) nitride complex that on hydrogenation, produces ammonia catalytically with the help of a rhodium hydride catalyst (Fig. 42). The rhodium catalysts promote H_2_ activation as well as hydrogen atom transfer in the catalytic transformation to produce free ammonia. The phenyl imine-based rhodium pre-catalyst was found to be more efficient than the phenyl pyridine-based pre-catalyst due to a higher tendency to facilitate PCET rather than hydride transfer pathways. In the case of the phenyl pyridine variant, using non-polar or non co-ordinating solvents can reduce the amount side reactions for better yield of NH_3_.^91^

In the current development strategies of metal complexes as catalysts for nitrogen fixation, rational ligand design and a better understanding of how it actually enhances the performance of the catalyst are of primary importance. It has been observed that PCP-type ligands consisting of N-heterocyclic carbene moiety as the carbon donor to bind to the metal center, work better as a catalyst for ammonia production, as explored by the Nishibayashi group, but not much work has been done with these types of ligands. Thus, in the coming future, the use of NHC-based ligands can prove to be a boost in the catalytic system as NHCs have the unique characteristic of binding strongly to the metal center. A specific strength of these compounds is their general capability to coordinate to the metal centers, extending from various electron abundant transition metals to electron-deficient main group metal cations and also high oxidation state metals. As NHCs can bind to metals with a high oxidation state, thus Mo would be a good choice. Thus, designing an NHC-based molybdenum catalyst might prove to be effective to convert dinitrogen into ammonia catalytically.

## 5. Conclusion and future prospect

Development and scale-up of ammonia production under ambient reaction condition are still in high demand. Catalytic synthesis of ammonia is a continuous process since the basic human need is food which requires nitrogen as a source. Ammonia is important raw material of urea. The high energy-intensive Haber Bosch process dominated the ammonia industry at the beginning of the 20th century. Developing alternative environment-friendly and energy benign techniques is of considerable importance. Minimizing the energy consumed in the currently existing technology for catalytic synthesis of ammonia is the main goal which, although in a slow pace, is being taken forward by implementing different strategies. Encouraging achievements have been made to obtain ammonia under mild reaction conditions, to name a few are electrocatalysis, photocatalysis, thermocatalysis, *etc.* Efforts have also been made to develop transition metal-bound homogeneous catalytic systems to turn N_2_ to NH_3_ catalytically. Rapid growth of organometallic catalysis has forecasted the achievement of ammonia preparation at ambient condition to be not very far. Blending of modern techniques like artificial intelligence (AI), machine learning (ML) with experimental findings may accelerate the alternative route of ammonia production at ambient conditions. Apart from fertilizer, ammonia is the potential starting material for hydrogen production. Therefore the tremendous research to achieve ammonia *via* energy saving methodology will be successful very soon and it is to be believed that organometallic catalysts will be the trump card in the upcoming technology.

## Author contributions

D. B. has collected the literature, prepared the manuscript, F. R. G. has prepared a few ChemDraw diagrams, wrote one segment of the review and B. S. has conceptualized, guided, and corrected the manuscript.

## Conflicts of interest

There are no conflicts to declare.

## Supplementary Material
